# New insights on Drug's design against candidiasis on the fructose biphosphate aldolase (Fba1) and the pyruvate kinase (Pk) of *Candida glabrata*

**DOI:** 10.1016/j.bbrep.2025.102175

**Published:** 2025-07-25

**Authors:** Edson E. Maqueda-Cabrera, Alejandro Castillo-Baltazar, Nancy A. Vázquez-López, Maritza Almanza-Villegas, María Teresa Ramírez-Apan, M. Carmen Ortega-Alfaro, José G. López-Cortés, Abel Moreno, Mayra Cuéllar-Cruz

**Affiliations:** a Departamento de Biología, División de Ciencias Naturales y Exactas, Campus Guanajuato, Universidad de Guanajuato, Noria Alta S/N, Col. Noria Alta, C.P. 36050, Guanajuato, Guanajuato, México; b Instituto de Química, Universidad Nacional Autónoma de México, Av. Universidad 3000, Ciudad Universitaria, Ciudad de México, C.P. 04510, México; c Instituto de Ciencias Nucleares, Universidad Nacional Autónoma de México, Av. Universidad 3000, Ciudad Universitaria, Ciudad de México, C.P. 04510, México

**Keywords:** *Candida glabrata*, Drug repositioning, Fba1, Pk

## Abstract

*Candida glabrata* is well known to be the second most common cause of invasive candidiasis (IC) within immunocompromised and hospitalized patients, after *Candida albicans*. *Candida* species adhere to host cells and implanted medical devices by means of cell wall proteins (CWP), of which the moonlight proteins have recently been described and are of particular importance because they have been identified in response to various virulence and/or pathogenic factors. Among the identified CWP moonlights, fructose-bisphosphate aldolase (Fba1) and pyruvate kinase (Pk) have been observed to confer immune protection against *C. albicans* and *C. glabrata* in a mouse model. In other pathogens, these proteins have been used as therapeutic targets. As the treatment of IC has been based on four main drug classes for decades, the *Candida* species has developed resistance mechanisms. In addition, *C. glabrata* has an innate resistance to the antifungal drugs, which makes the treatment of IC by this pathogen difficult. It is essential to have new formulations that allow new treatments of patients affected by this pathogen, so new targets with antifungal activity is of primary necessity. For this purpose, in this study we propose the moonlight CWPs Fba1 and Pk as novel candidates for drug targets. Using structural modeling, virtual database analysis, *in vitro* susceptibility tests, and enzymatic activity assays, we propose the use of new chemical molecules as potential antifungals against *C. glabrata*. In this sense, we chose to evaluate three chemical molecules (FE1, FE2 and FE3), whose chemical structure gives them the possible molecular leadership against Fba1 and Pk. Through the susceptibility experiments, our data showed that of the three molecules evaluated, FE1 was the best ligand against *C. glabrata*. We also found that Fba1 and Pk of *C. glabrata* had the characteristics of therapeutic targets against IC. In the present work, considering a group of tools *in silico* and experiments *in vitro* it was possible to identify the best candidate molecule as a possible antifungal for the treatment of IC caused by *C. glabrata.*

## Highlights

• *Candida glabrata* is the second most common cause of invasive candidiasis (IC) in immunocompromised and hospitalized patients.
• Among the identified CWP, Fba1 and Pk have been observed to confer immune protection against *C. glabrata* in a mouse model.
• Fba1 and Pk have been used as therapeutic targets.
• *C. glabrata* has an innate resistance to the antifungal drugs, which makes the treatment of IC by this pathogen difficult.
• In this study we propose the moonlight CWPs Fba1 and Pk as novel candidates for drug targets.

## Graphical abstract

**Figure:**
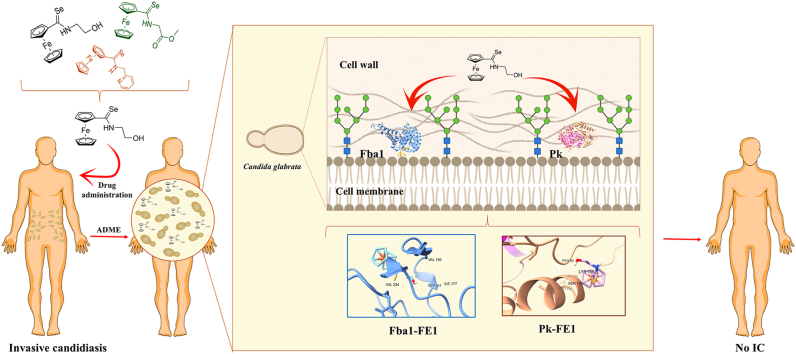

## 1 Introduction

Infectious illnesses caused by fungi are of special relevance because of the high mortality rates of hospitalized and immunocompromised patients. Recently, the World Health Organization classified the priority fungal pathogens in groups: critical, high and medium [1,2]. *Candida* pathogen species are found in the three groups, evidence that confirms that these fungi are responsible for a considerable percentage of invasive fungal infections (IFI) in hospitals [2,3]. It has been reported that approximately 1,565,000 persons per year suffer an IFI by *Candida*; 995,000 dies, which represents a mortality rate of 63.6 % [4], with *C. albicans* as the most prevalent, followed by *C. glabrata* [5]. Recently, it has been proposed that *C. glabrata* be named as *Nakaseomyces glabrata* [6] or *Nakaseomyces glabratus* [7]. However, other authors have carried out an evaluation of the medical and clinical implications that this name change implies [8], and for this reason, we will use the name *C. glabrata* in the present work. The ability of *Candida* to invade various organs, as well as implanted medical devices in the human host is due to its virulence and pathogenic factors [9]. We find that adhesion, resistance to attack by macrophages and the resistance to oxidative stress are among the pathogenicity factors of these fungi [10,11]. The virulence factors include the secretion of proteinases, the morphologic changes, the phenotypic changes and the formation of biofilms [12]. Of these factors, adhesion is the first step for *Candida*'s colonization of the host, and it is an essential step for the establishment of infection [13]. The *Candida* species adhere to the cells of the host and to the implanted medical devices in the host via the cell wall proteins (CWP) [14] *Candida* possesses several classes of CWP: the first class are the proteins which anchor a glycophosphatidylinositol (GPI) [15,16]. The second class are the peptides with internal repeats (Pir), which are found covalently united to the β-1,3 glucans [17]. The third class of proteins lack a covalent bond to the polysaccharide matrix of the CW [18] as are the recently described moonlight proteins [19,20]. These are of special relevance because they have been identified in response to oxidative stress (OSR), nitrosative stress, formation of biofilms and adherence to various medical devices [19,20]. Additionally, it has been identified that the sera of candidiasis patients recognize CWP moonlight type; some are non-virulent immunogenic and not ubiquitous during an infection by *C. albicans* [21]. In a dot blot study, it was suggested that these CWPs are possibly the main immunogens during infections by *C. albicans* [21]. Of the CWP moonlight type enzymes that are identified in common in the various pathogen species of *Candida* in response to the various virulence and pathogenic factors fructose-bisphosphate aldolase (Fba1) and pyruvate kinase (Pk) were found. In a murine model, these two proteins have been shown to generate immune protection to rats against *C. albicans* and *C. glabrata* [22]. In other pathogens, these proteins have been used as therapeutic targets, as is the case of Fba1 that has been identified in the serum of patients with candidemia [23]. Pk has been proposed as a therapeutic target against *Leishmania* species [24], *Staphylococcus aureus*, Methicillin-sensitive *S. aureus* (MSSA), *Listeria monocytogenes*, *Enterococcus faecium*, *Enterococcus faecalis*, multidrug-resistant (MDR) MRSA, *Staphylococcus saprophyticus*, *Staphylococcus haemolyticus*, *Staphylococcus epidermis*, *Acinetobacter baumannii*, *Klebsiella pneumoniae*, *Escherichia coli* DYW330, *Pseudomonas aeruginosa* PAO1 and *Pseudomonas aeruginosa* PAO75 [25,26]. In this way, Fba1 and Pk have been shown to play an important role both in the metabolism as well as in the pathogenesis of various pathogenic microorganisms, including the *Candida* species. The treatment against IC for decades has been based almost exclusively using four classes of antifungal drugs such as polyenes, azoles, echinocandins and the analog of pyrimidine 5- flucytosine [27], and this has caused resistance mechanisms by *Candida* against them, which results in patients with various episodes of recurrent candidiasis and eventually to death [28]. In addition, *C. glabrata* presents innate resistance to the antifungals based on azole, which makes IC treatment of this pathogen difficult [29]. In this context, it is crucial to have new drugs that allow offering new treatments for IC to *C. glabrata* patients, which would favor the reduction of mortality associated with this pathogen. Therefore, the identification of new antifungal targets based on unique mechanisms of action are of essential necessity. For this, the CWPs moonlight Fba1 and Pk, due to their therapeutic targets on other pathogens and their identification in *Candida* species to various virulence and pathogenicity factors, are proposed for the first time by us in this work as CWP novel drug target candidates in the treatment of *C. glabrata*. This is because, at present, there is no specific treatment against this pathogen, which has led to high rates of morbidity and mortality in patients suffering this type of *Candida*. Using structural models, analysis of virtual databases and of assays of enzymatic activity, we propose the use of chemical molecules as possible antifungals against *C. glabrata*. In this sense, we chose to evaluate three chemical molecules (FE1-FE3) whose chemical structure allows them characteristics of possible leadership potentials in the treatment against Fba1 and Pk. Our data shows by susceptibility experiments that of the three evaluated molecules, FE1 proved to be that is the best ligand against *C. glabrata.* We also determined that *C. glabrata*'s Fba1 and Pk possess the characteristics needed for being therapeutic targets against IC.

## 2 Materials and methods

### 2.1 Bioinformatic analysis

#### 2.1.1 Protein sequence retrieval

The sequences for the *C. albicans* and *C. glabrata* proteins were retrieved in FASTA format from the National Center for Biotechnology Information (NCBI, accessed May 2024). Among the selected entries, those corresponding to the most complete and representative sequences were selected, while redundant or truncated isoforms were excluded. The corresponding accession numbers were: i) CaFba1 ID:XP_722690.1, CgFba1 ID: KTB27082.1 ii) CaPk ID: XP_714934.1, CgPk ID: XP_449865.1 [30].

#### 2.1.2 Ortholog identification

Pairwise BLASTp searches were performed using the *C. glabrata* sequences as queries against the non-redundant protein database (NCBI, 2024) to identify potential human orthologs. The searches were conducted on the NCBI BLAST server with the following parameters: E-value threshold ≤ 1e-5, substitution matrix: BLOSUM62 and filtering enabled for low-complexity regions (default settings).

Sequences with high identity, high query coverage, and low E-values (closer to zero) were prioritized as indicative of significance. To ensure ortholog assignment, candidate human sequences of Pk were analyzed using the EggNOG v5.0 database [31] (http://eggnog5.embl.de/#/app/home), which integrates phylogenetic and functional annotations. This confirmed the human ortholog HsPk ID: NP_000289.1, retrieved in FASTA format from NCBI. In contrast, for the *C. glabrata* Fba protein (a type II aldolase), no human ortholog was identified, as humans lack type II aldolases. Instead, a functional analog was considered, HsFba1 ID: CAA30270.1, which catalyzes the same reaction but is evolutionarily unrelated.

#### 2.1.3 Multiple sequence alignment and domain identification

Multiple sequence alignments of the fungal and human proteins were carried out using the Clustal Omega server v1.2.4 [32,33] (https://www.ebi.ac.uk/jdispatcher/msa/clustalo). Input sequences were uploaded in FASTA format, ClustalW was selected as the output format and the resulting Percent Identity Matrix was downloaded in plain text format. Protein domain identification was performed using the InterPro server [34] (https://www.ebi.ac.uk/interpro/). Each sequence was submitted via the interface using default parameters, and the predicted domains were recorded for comparative analysis.

### 2.2 Three-dimensional (3D) protein structure modeling

The monomeric 3D structure of Fba1 and Pk was predicted using AlphaFold3 [35]. The corresponding FASTA sequences were uploaded, and the structures were predicted using the default settings of AlphaFold3. This version was selected because it provides more accurate structural information (including cofactors and metal ions) compared to previous versions.

#### 2.2.1 Metal ion incorporation

For Fba1, a metal ion was manually incorporated based on the crystal structure of *C. albicans* (PDB: 6LNK), which includes annotated cofactor-binding sites. The coordinates of the metal ions were transferred to the predicted model, and Gasteiger charges were assigned using UCSF ChimeraX [36]. This manual incorporation was chosen because a high-quality crystallographic template with clearly annotated cofactor-binding sites was available, allowing for a more accurate representation of the metal-binding regions. In contrast, for Pk, no suitable crystallographic templates with annotated cofactors were available. Therefore, AlphaFold3 was used to predict the structure, including cofactor binding, leveraging its advanced integrated cofactor modeling capabilities. AlphaFold3 [35] was selected for Pk due to its ability to model cofactor interactions, offering an improvement over traditional methods when appropriate templates are lacking.

#### 2.2.2 Structural quality assessment

The predicted models were evaluated using SAVES v6.0 (https://saves.mbi.ucla.edu), which integrates the following tools: ERRAT v2.0: overall quality factor [37]; Verify-3D v1.0: 3D/1D profile compatibility [38], and PROCHECK v3.5.4: Ramachandran plot analysis [39]. The structural quality analysis was performed every time a modification was made to the protein models to verify optimizations, considering thresholds and manually inspecting Ramachandran plots to ensure that the structural geometry was within acceptable parameters.

#### 2.2.3 Loop refinement and targeted correction

Loop refinement was performed using ModLoop [40] (https://modbase.compbio.ucsf.edu/modloop/) to relieve strain and improve geometry in flexible regions of the model. Specific residues, 301–303 in *C. albicans* Pk, 298–300 in *C. glabrata* Pk, and 339–342 and 20–23 in *H. sapiens* were selected for refinement, based on prior low scores in previous analysis. This refinement led to improvements in ERRAT and Verify-3D scores, as well as improved model fit. No refinement was required for Fba1 proteins.

#### 2.2.4 Energy minimization

The top-scoring models were submitted to the YASARA Energy Minimization Server to improve intramolecular interactions. The minimization was carried out using the AMBER14 force field with default parameters (up to 5000 steps or until convergence). The minimized models were analyzed using the SCORE function in YASARA [41] to confirm energy reduction.

### 2.3 Modeling of the ligands

The chemical structures of FE-FE3 were previously reported in earlier work and were used here as input structures for 3D modeling and docking studies.

The initial chemical structures were created using ChemDraw, where each compound was drawn in its two-dimensional (2D) form. These 2D representations were saved in CDXML format. The CDXML files were imported into Avogadro [42], an open-source molecular editor selected for its user-friendly interface and integrated tools for 3D structure generation and energy minimization. In Avogadro, the three-dimensional (3D) conformations were automatically generated and subsequently subjected to energy minimization using the Universal Force Field (UFF), chosen for its broad parametrization and suitability for small organic molecules, including those with uncommon atoms or functional groups. Minimization was performed using the conjugate gradient algorithm, with no constraints applied, a maximum of 500 steps, and a convergence criterion of 10^−7^ kcal/mol. This convergence was achieved for all ligands. The minimized structures were saved in PDB format for further use in molecular docking studies.

### 2.4 Molecular docking

Molecular docking studies were conducted using AutoDock Vina 1.1.2 [43] through the PyRx 0.8 interface [44]. Protein and ligand structures were initially imported in PDB format and converted to PDBQT format within PyRx. Proteins were defined as rigid macromolecules, while ligands were treated as flexible, with rotatable bonds automatically assigned by the software.

#### 2.4.1 Protein preparation

Protein models were prepared using YASARA. Water molecules and non-essential heteroatoms were removed, and polar hydrogens and partial atomic charges were added. Protein torsions were kept rigid, consistent with the rigid receptor model employed by AutoDock Vina. The protein was placed in an explicit aqueous environment to validate structural integrity, but docking was performed in vacuum conditions within PyRx.

#### 2.4.2 Ligand preparation

Ligands (FE1-FE3) were previously energy-minimized in Avogadro using the Universal Force Field (UFF). In PyRx, torsional flexibility was automatically assigned to all rotatable bonds, protonation states were kept at physiological pH (7.4), and Gasteiger charges were assigned by default during the PDBQT conversion.

#### 2.4.3 Docking setup and parameters

A blind docking approach was used to allow unbiased exploration of the entire receptor surface. The exhaustiveness parameter was set to 8. AutoDock Vina uses a stochastic global optimization method (iterated local search) to identify the most favorable ligand-receptor binding poses based on predicted binding free energy.

For CgFba1:

Grid center: X: 1.1934, Y:1.5665, Z: 0.7670.

Grid size: X:66.8549 Å, Y:51.3813 Å, Z: 61.6638 Å.

For CgPk:

Grid center: X:29.5736, Y:0.6910, Z:21.3161.

Grid size: X:50.0071 Å, Y: 68.2179 Å, Z: 75.6716 Å.

The best binding poses were selected based on the lowest binding free energy values reported by AutoDock Vina.

#### 2.4.4 Post-docking analysis

Docked complexes were visualized using UCSF ChimeraX [40] for three-dimensional (3D) analysis and BIOVIA Discovery Studio Visualizer 2024 for two-dimensional (2D) interaction mapping. Interaction types such as hydrogen bonds, hydrophobic interactions (alkyl), π systems and electrostatic interactions were identified to assess ligand-protein binding quality.

### 2.5 Expression and purification of the Fba1 and Pk proteins

#### 2.5.1 Induction of the recombinant proteins

The recombinant protein Fba1 was cloned from the BL21 strain of *E. coli*, while Pk was cloned from the PKJE7 strain. A colony of each of the transformed strains was taken and inoculated into 30 mL of liquid LB medium (casein peptone 1 %, yeast extract 0.5 %, and NaCl 1 %). For the growth of the Fba1 strains, supplements were made with ampicillin 100 μg/mL, while the Pk strain was supplemented with chloramphenicol 25 μg/mL and incubated at 37 °C for 12 h with constant stirring. Subsequently, 500 mL of the LB medium supplemented with the corresponding antibiotic was inoculated with 30 mL of preinoculum and incubated at 37 °C with constant stirring until the (OD_600nm_) of 0.5–0.8 optical density was reached. A 1.0 mL aliquot was taken (non-induced control) and IPTG at a concentration of 1 mM was added to the rest for 4 h. After the induction, 1 mL (induced control) was taken and the rest of the culture was centrifuged at 10,000 g for 10 min at 4 °C, the supernatant was discarded, and the pellet was preserved. The non-induced controls and the induced were centrifuged in the same conditions as the rest of the induced culture. Both the cellular pellet of the controls and of the induced culture were stored at −20 °C.

#### 2.5.2 Extraction of proteins

The cellular pellets were resuspended in a minimum volume of lysis regulating solution (Na_2_HPO_4_ 50 mM, NaCl 500 mM, imidazole 5 mM, and PMSF 1 mM) at pH 8.0. The resuspended pellets were lysed by ultrasound with 5 s of sonication and 5 s of rest for 8 min, at an amplitude of 30, keeping the extracts in an ice bath. After this, the cellular lysate was centrifuged at 14,000 g for 20 min at 4 °C, and the supernatant was recovered, which contained the recombinant proteins Fba1 or Pk.

#### 2.5.3 Purification of the recombinant proteins

The recombinant proteins were purified by liquid chromatography of proteins at high speed, FPLC (Cytiva, ÄKTA™ Go), by affinity chromatography. HisTrap HP 1 mL columns were used, which were washed with 5 column volumes (CV) of distilled water. To balance the column, 5 VC of elution buffering was used and then 10 VC of binding buffering solution, at a constant flow of 1 mL/min and a pressure of 0.50 MPa. A 1 mL of protein extract was injected and passed through the column with 5 CV of binding buffering solution later to be treated with a lineal gradient of 15 VC of elution buffering solutions at a constant flow of 1 mL/min and at 0.50 MPa pressure. Fractions of 1 mL were collected in Eppendorf microtubes of 1.5 mL.

#### 2.5.4 SDS-PAGE of the fractions obtained in the purification

The fractions obtained in the purification were evaluated via electrophoresis in a polyacrylamide gel at 12 % (w/v). The electrophoresis was carried out at 90 V for 30 min, carrying out the rest of the process at a constant voltage of 120 V for 1 and a half hours. The gel was stained using Coomassie blue (Fig. S10).

#### 2.5.5 Quantification of the proteins

The concentration of the pure proteins was determined with the Lowry method [45].

### 2.6 Obtaining the chemical compounds

#### 2.6.1 Synthesis and characterization

The ferrocenylselenoamides, FE1, FE2 and FE3 were obtained following the procedure reported by Lopez-Cortés et al. [46,47].

General procedure for the synthesis of ferrocenylselenoamides.

a) Preparation of selenating mixture: 10 mL of ethanol were poured into a suspension of 0.023 mol of NaBH_4_ divided in 4 parts of 0.023 mol of powdered selenium. After mixing for 30 min at room temperature under rigorous stirring. This was performed under a nitrogen atmosphere till no hydrogen production was observed.
b) Synthesis of ferrocenylselenoamides (FE1, FE2): The corresponding amine (4.8 mmol) was poured into 2.3 mmol ferrocenyl Fischer ethoxycarbene complex in 20 mL of anhydrous diethyl ether under a nitrogen atmosphere. The reaction mixture was stirred at room temperature for 45–180 min and then diluted with 20 mL of water. The isolated organic phase was separated and dried with anhydrous Na_2_SO_4_, then, using a vacuum system, the solvent was carefully extracted. The raw product suspended in a minimum amount of ethanol was added to the selenated reagent previously described in (a). TLC on silica gel was performed to monitor the status of the reaction. After completing the reaction, the solvent was extracted under a vacuum system having a NaClO trap. Then the remaining mix was dissolved in water, and the product was extracted with CH_2_Cl_2_ and then dried with anhydrous Na_2_SO_4_. When evaporating the solvent, the raw product was purified using a silica-gel column at different hexane ratios (using ethyl acetate as eluent).

***N*-(2-hydroxyethyl)ferroceneselenoamide (FE1):**^1^H NMR (300 MHz, DMSO-*d*_6_) δ = 10.01 (s, 1H), 5.11–5.07 (m, 2H), 4.87 (t, *J* = 5.2 Hz, 1H), 4.53–4.48 (m, 2H), 4.15 (s, 5H), 3.80 (t, *J* = 5.4 Hz, 2H), 3.72 (t, *J* = 5.4 Hz, 2H). ^13^C NMR (75 MHz, DMSO) δ = 201.28, 86.00, 71.63, 71.18, 70.43, 58.57, 51.39.

**Methyl (ferrocenylcarbonoselenoyl) glycinate (FE2):**^1^H NMR (300 MHz, DMSO-*d*_6_) δ = 10.35 (s, 1H), 5.08 (s, 2H), 4.55 (d, *J* = 10.8 Hz, 4H), 4.24 (s, 5H), 3.70 (s, 3H). ^13^C NMR (75 MHz, DMSO) δ = 204.29, 168.52, 85.02, 72.13, 71.25, 70.46, 52.40, 49.98.

***N*-(pyridin-2-methyl)ferroceneselenoamide (FE3):**^1^H NMR (300 MHz, CDCl_3_) δ = 9.68 (s, 1H, H-8), 8.62 (d, *J* = 4.8 Hz, 1H, H-14), 7.75 (s, 1H, H-12), 7.38 (s, 1H, H-11), 7.32–7.27 (m, 1H, H-13), 5.00 (s, 4H, H-2, 3), 4.54–4.46 (m, 2H, H-9), 4.22 (s, 5H, H-1). ^13^C NMR (75 MHz, CDCl_3_) δ = 201.92 (C-5), 153.99 (C-10), 148.76 (C-14), 136.93 (C-12), 122.68 (C-13), 122.12 (C-11), 86.30 (C-4), 71.32 (C-2), 70.78 (C-1), 69.38 (C-3), 52.55 (C-9).

All compounds (from FE1to FE3) are >95 % pure by HPLC analysis. HPLC traces for all compounds are included in the supporting information (Figs. S1–S9).

### 2.7 Enzymatic activity

The enzymatic activity was carried out starting from the purified enzymes, which were incubated with their respective substrate at 60 °C for 20 min using buffering solutions (HEPES 50 mM, KCl 100 mM, MgCl_2_ 10 mM, ADP 1.5 mM, Glycerol at 10 % (v/v) and Triton X-100 at 0.1 % (v/v), pH 7.0 for Fba and Pk, as well as a concentration of 10 mM of Zn^2+^ for Fba1 and 10 mM of Mn^2+^ and 5 mM of K^+^ for Pk. To evaluate the effect of the FE1 compound on the enzymatic activity, the previously described procedure was carried out for each enzyme, adding the corresponding concentrations of 1.0, 2.5, 5.0, 7.5, and 10 μM of FE1. The absorbances were read at 340 nm in the UV–Visible Epoch spectrophotometer, BioTek Instruments, Inc., supported by the Gen5TM All-In-One Microplate Reader software. Each experiment was carried out six times and the statistical analysis of the data was performed with GraphPad Prism 9 software.

### 2.8 Pharmacokinetic and toxicological analysis

Two complementary computational tools, SWISS-ADME [48] and pkCSM [49], were used to evaluate the pharmacokinetic properties and potential toxicity of the ferrocene-based compounds (FE1-FE3).

#### 2.8.1 Prediction of pharmacokinetic properties and drug likeness

The SWISS-ADME server [48] was employed to predict physicochemical properties, pharmacokinetic behavior (intestinal absorption, blood-brain barrier permeability, CYP450 inhibition), and drug-likeness criteria (Lipinski). The compounds were submitted in SMILES format, generated from the previously built molecular models. Output parameters included logP, topological polar surface area (TPSA) and number of rotatable bonds, among others.

#### 2.8.2 Toxicity prediction

Toxicological properties were predicted using the pkCSM server [49], which applies graph-based signatures to estimate various toxicity endpoints. The compounds were submitted in SMILES format and the output parameters included AMES toxicity (mutagenicity), acute toxicity (LD50 in rats), hERG inhibition (cardiotoxicity) and hepatotoxicity.

### 2.9 Susceptibility assays by disk diffusion technique

An overnight culture of *Candida glabrata* was prepared by inoculating a single colony using a bacteriological loop into 5 mL of YPD broth (1 % yeast extract, 2 % peptone, 2 % dextrose) and incubating at 28 °C with constant agitation at 120 rpm. The optical density of the culture was measured at 600 nm and adjusted to 0.5 O.D. units using sterile deionized water, corresponding approximately to 1x10^6^-10^7^ CFU/mL based on established correlations for yeast. A volume of 75 μL of the adjusted inoculum was evenly spread onto YPD agar plates (same composition as the broth medium with 2 % agar) using a sterile Drigalski spatula. Sterile 6 mm Whatman No. 40 filter paper disks were placed equidistantly on the agar surface using sterile forceps. Every disk was loaded with 50 μL of the test compound solution at 17 mg/mL (corresponding to 850 μg per disk). Disks containing only solvent (DMSO) were used as negative controls, while disks loaded with nystatin served as positive controls to validate assay performance. Plates were incubated at 28 °C for 24 h, after which the diameters of the zones of inhibition were measured in millimeters. The diameter of the DMSO inhibition halo (negative control) was subtracted from all others to represent only the effect of the compounds. All treatments were performed in triplicate (n = 3), and results were expressed as the mean ± standard deviation.

### 2.10 Cytotoxicity assay

The cytotoxicity of the FE1 compound was evaluated *in vitro* using two cell lines: HaCat (human keratinocytes) and COS-7 (African green monkey kidney fibroblasts). Cells were cultured in Dulbecco's Modified Eagle Medium (DMEM) supplemented with 10 % fetal bovine serum (FBS), 2 mM l-glutamine, 1 % non-essential amino acids, and 1 % penicillin-streptomycin (Gibco). The cells were maintained at 37 °C in a humidified atmosphere with 5 % CO_2_.

Cell viability was assessed using the trypan blue exclusion method and counted manually with a Neubauer hemocytometer. Only cultures with viability greater than 95 % were used for seeding. Cells were trypsinized and resuspended in fresh medium to a final concentration of 5 × 10^^4^ cells/mL, and 100 μμL of the suspension (equivalent to 5000 cells/well) was seeded into 96-well flat-bottom plates (Costar) and incubated for 24 h to allow cell attachment. This concentration was selected based on previous studies and the characteristics of the cell lines, ensuring adequate cell attachment and providing a reliable dose-response curve while maintaining an optimal signal-to-noise ratio for absorbance readings.

A pure solution of FE1 was prepared at a concentration of 20 mM in DMSO. From this stock, an intermediate solution of 200 μM was prepared in sterile distilled water (final volume of 1 mL). This dilution resulted in a final DMSO concentration of 1 %. Working solutions were prepared from the intermediate solution (200 μM) by dilution in DMEM, yielding final concentrations of 2, 6.2, 11.2, 20 and 36 μM.

After incubation, cells were treated with 100 μL of each working solution, resulting in final concentrations of 1.0, 3.1, 5.6, 10 and 18 μM, with corresponding final DMSO concentrations ranging from 0.005 % to 0.09 %. Each treatment was performed in triplicate (n = 3). In addition, vehicle-only (DMSO 0.09 % v/v) and untreated cells were included as negative controls. Cells were incubated with the compound for 48 h, after which cell fixation was performed by the gentle addition of 50 % cold trichloroacetic acid (TCA, 50 μL/well), and plates were incubated at 4 °C for 1 h. Plates were then washed with distilled water and air-dried. Staining was conducted using 0.4 % sulforhodamine B (SRB) in 1 % acetic acid (100 μL/well) for 30 min at room temperature. Excess dye was removed by washing the cells three times with 1 % acetic acid, followed by air drying. The protein-bound dye was solubilized with 10 mM unbuffered Tris base (100 μL/well), and plates were agitated on a lab shaker for 5 min. The absorbance was read at 515 nm (Bio-Tek Instruments), because according to Warawdekar et al. [50], it optimizes the assay and improves the reliability and precision of the results. The results were analyzed as the percentage of viable cells relative to untreated controls.

### 2.11 Determination of minimum inhibitory concentration (MIC) of FE1

This antifungal susceptibility test was performed using the broth microdilution method, according to the standardized protocol of the Clinical and Laboratory Standards Institute (CLSI), as outlined in document M27-A3 [51]. Briefly, a 24-h culture of Candida glabrata grown in YPD medium was used to prepare a pre-inoculum, which was adjusted to an optical density of 0.5 in sterile saline solution (0.85 %). Subsequently, a 1:1000 dilution was made in RPMI 1640 supplemented with glutamine and without sodium bicarbonate (Gibco), adjusted to pH 7.0 and containing 0.2 % glucose, which served as the working solution.

The compound FE1, which is water-insoluble, was dissolved in DMSO at a concentration 100 times higher than the maximum concentration to be tested (6.4 mg/mL). A 1:50 dilution with RPMI medium was then performed, resulting in an antifungal concentration twice the desired final concentration and a DMSO concentration of 2 % (128 μg/mL). From this stock solution, two-fold serial dilutions were prepared (with RPMI medium) to obtain the desired concentrations in separated tubes (128–0.25 μg/mL). In a 96-well microplate, 100 μL of each FE1 solution was added to the corresponding wells, followed by 100 μL of the working inoculum solution (having final concentrations to evaluate of 64-0.125 μg/mL). The plate was incubated at 35 °C for 24 h without shaking, and the optical density was measured at 530 nm. The results were compared against a negative control (uninoculated RPMI medium with a DMSO concentration of 1 %) and a positive control (working inoculum solution without antifungals).

### 2.12 Statistical analysis

A Shapiro-Wilk test was carried out for each evaluated group to determine the distribution of the data and to carry out an analysis of variance (ANOVA) or Kruskal-Wallis test to see if the data was parametric. A significance level of p < 0.05 was considered statistically significant. When significant differences were found, Tukey's post hoc test (for ANOVA) or Dunn's multiple comparisons test (for Kruskal-Wallis) was used to identify pairwise differences between treatment groups.

## 3 Results and discussion

### 3.1 The *in vitro* experiments show that FE1 is the best ligand against *C. glabrata*

**Fig. 1:**
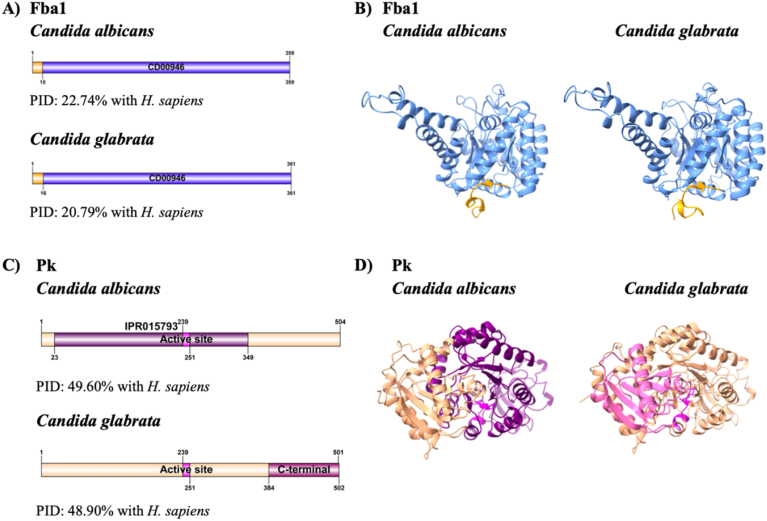
**Bioinformatic analysis of Fba1 and Pk of *C. albicans*, *C. glabrata* and *H. sapiens*.** Illustration of the domains of A) Fba1 and C) Pk obtained with the InterPro data base. Modeled 3D structures by AlphaFold2 of B) Fba1 and D) Pk.

For the purpose of evaluating whether the CWP moonlight types Fba1 and Pk of *C. albicans* and *C. glabrata* might be able to be proposed as possible therapeutic targets for the design of new chemical molecules with antifungal potential, the first step was to choose the sequence of amino acids to obtain the tridimensional (3D) structure of the three proteins. For Pk, the crystallographic structure has not yet been reported. When carrying out the amino acid sequence of Fba1 and Pk of *C. albicans*, *C. glabrata,* and *H. sapiens* with BLAST in the UNIPROT database, it was found that in the case of Fba1 of *C. albicans* and *C. glabrata* that these sequences share an identity percentage of 22.74 % and 20.79 %, respectively, compared to Fba1 of *H. sapiens* (Fig. 1A). Fba1 for *Candida* presents a domain CD00946, and for *H. sapiens,* a domain PF00274. While in the case of the Pk of *C. albicans* or *C. glabrata*, there is a 49.60 % and 48.90 % of identity in comparison with the Pk of *H. sapiens* (Fig. 1C). These results show that the Fba1 and Pk of *C. albicans* or *C. glabrata* present a percentage of identity that varies from 20 % to 60 % regarding the Fba1 and Pk of *H. sapiens*. This fact is relevant because a change of 10 % in identity among proteins is considered to represent an evolutionary distance of many years. Therefore, a change of 80 %–70 % in identity might reflect a divergence of 200 million years before in time, but the change from 30 % to 20 % might mean a temporal divergence of a thousand million years [52]. Notably, while Pk is considered an ortholog (implying a common evolutionary origin), Fba1 is categorized as an analog, likely reflecting functional similarity without direct evolutionary relatedness. This distinction is important because, in the case of Fba1, the low sequence identity supports the hypothesis of significant structural and evolutionary divergence from the human enzyme. For Pk, although it is an ortholog, the moderate-to-low identity still suggests sufficient divergence that could be exploited therapeutically. In this way, the fact that there is such a big difference among Fba1 and Pk of *C. albicans* or *C. glabrata* with regard to the proteins of *H. sapiens* indicates that the CWP moonlight type Fba1 and Pk of *C. albicans* or *C. glabrata* are ideal candidates as therapeutic targets, as has been reported in other pathogens [[24], [25], [26]], without harmful effects on the human host. Once the alignment of Fba1 and Pk was carried out, the modeling of the 3D structures by the online platform AlphaFold3 was performed. It was decided to use AlphaFold because this technique is a known 3D protein structure whose sequence is similarly modeled [[53], [54]]. This similarity makes it possible to align both sequences and reconstruct the target protein's most probable structure, using the 3D structure of the protein known as mold and reported in the Protein Data Bank (PDB), which results in more trustworthy structures.

The results of the modeling of Fba1 of both *C. albicans* and *C. glabrata*, as well as that of *H. sapiens*, showed a representative domain in the InterPro analysis (amino acids 16–361) made up of beta pleated, alpha-helix and loops (Fig. 1D). The Pk are homotetramers of identical subunits, each of which is found made up of three or four domains that correspond to the N-terminal, as well as domains A, B and C [[55], [56], [57], [58]]. The active site is found in the interface of domains A and B, and the allosteric site is located in domain C [[55], [56], [57], [58]]. The modeling of the Pk monomers displays the domain IPR015793 (amino acids 23–349) formed by beta sheets, alpha-helixes and loops for *C. albicans* and *H. sapiens*, as well as the C-terminal domain (amino acids 384–502) with the previously mentioned structural motifs for *C. glabrata* and *H. sapiens*, as well as the active site (amino acids 239–251, Fig. 1C).

**Fig. 2:**
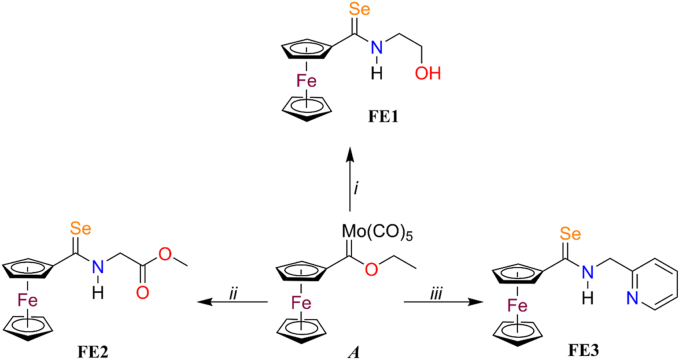
**Chemical molecules used as possible new drugs against the Fba1 and Pk proteins of *C. glabrata***. *i*) See ref. 46: 1) Ethanolamine, ether, 2) Se/NaBH_4_, Ethanol; *ii*) 1) 2-Picolylamine, CH_2_Cl_2_, 2) Se/NaBH_4_, Ethanol; *iii*) See ref. 47: I_2_, Pyridine.

Once the 3D structures of Fba1 and Pk of *C. albicans*, *C. glabrata,* and *H. sapiens* (Fig. 1) were obtained, we confirmed that there is a difference between the proteins of one species and the other. The chemical molecules containing chemical groups with possible antifungal potential against *C. glabrata* was identified. The identification of new chemical molecules that interact with these three proteins of *C. glabrata* is fundamental because even though we have an array of available antifungals for commercial use, the mortality rates for patients with IC have increased considerably in the last decades [59]. This is due in part to the fact that the antifungal most commonly prescribed to patients is based on the azole group [60], which is effective against *C. albicans.* However, these drugs are neither as effective on non-*C*. *albicans Candida* species (NCAC) nor on *C. glabrata,* as these species present innate resistance to the azole compounds [61]. The high rates of mortality of patients associated with *C. glabrata* increase annually because the drugs used against *C. albicans* are being designed without considering the NCACs, such as *C. glabrata*. For this reason, it is of foremost concern to rely on novel drugs against this species that will provide adequate treatment to patients with IC *C. glabrata* patients, enhancing their quality of life. These medications could reduce or even avoid the pain and discomfort of this illness, as well as prevent recurrent and combined treatments. This would then lead to a reduction in mortality associated with IC due to this pathogen. Identifying chemical molecules that interact with the proteins that are possible therapeutic targets is of outmost importance for developing new drugs [61]. In this way, one of the present work's objectives is to identify the chemical molecules that interact with the Fba1 and Pk of *C. glabrata*. It is reported that the first step in the development of drugs is the identification of compounds that are bound to be the therapeutic target or that show biological activity in an *in vitro* assay. Over time, several strategies have been developed to identify new chemical molecules that might be used as therapeutic targets. Among these strategies for discovering new potential drugs are *in vitro* assays and high-throughput screenings. High-throughput screening (HTS) is the conventional way to select new chemical compounds. This involves carrying out experiments in the laboratory, where libraries of compounds are analyzed. However, there are some limits to this, such as budget, infrastructure, time, and variability in the results obtained [[61], [62], [63]]. Recently, the design or identification of new chemical molecules as possible drugs assisted by computer presents advantages, such as a first screening because the time of analysis of inclusion or discrimination of compounds is considerably reduced, not great physical infrastructure is required, the selection of candidate molecules is improved, as is the optimization of the leading molecules to improve desired properties and it also decreases the adverse effects. Another strategy to obtain bioactive chemical molecules is through the repositioning of drugs, in which drugs that were used for the treatment of one illness are repositioned to be used for another illness, with new indications that vary from the original [[64], [65], [66], [67]]. In this sense, we chose to evaluate a group of three chemical compounds (FE1-FE3), including a ferrocene imidazole[1,5*a*]pyridine and some precursors for obtaining it (Fig. 2). The structural template could confer properties as possible, leading molecules against the Fba1 and Pk proteins of *C. glabrata.* These molecules were originally designed as potential anti-cancer candidates [46].

As can be seen, all compounds possess the ferrocene group, which could enhance their biological potential as bioactive molecules [68]. Additionally, the antibacterial effect of the ferrocene derivatives has been attributed to the formation of ferrocenium, which results in the generation of ROS, specifically the hydroxyl radical [69]. Furthermore, FE3 contains a pyridine to improve the physicochemical properties of the candidate. This nitrogen heterocycle is commonly found in antifungal drugs [70,71]. Ferrocene hybrid drugs are one of the most promising strategies for developing new antimicrobial agents because they act on multiple objectives and can reduce the capability of the microorganisms to develop resistance [69]. The synthesis of FE1-FE3 begins with the previously reported ferrocenyl Fischer carbene *A* [72]. This compound was transformed into the corresponding selenoamides using a sequential, two-step procedure involving the synthesis of the corresponding aminocarbene using different amines (ethanolamine for FE1, glycine methyl ester for FE2, and picolylamine for FE3) and selenative demetallation of these organometallic compounds developed previously by López-Cortés et al. [46,47].

**Fig. 3:**
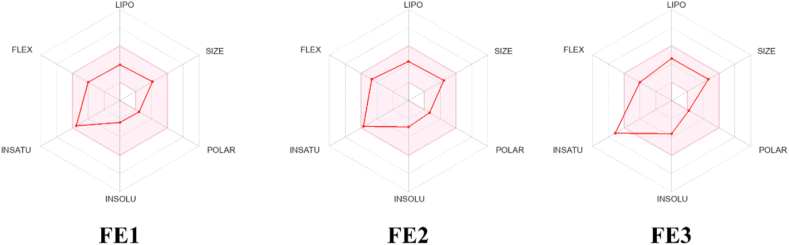
**Diagram of the bioavailability of the FE1 to FE3 chemical molecules. The area outlined in red represents the optimal interval of the evaluated parameters.** LIPO: liposolubility; SIZE: size; POLAR: polarity; INSOLU: insolubility; INSATU: insaturation; FLEX: flexibility.

Once the molecules F1–F3 were chosen against the Fba1 and Pk of *C. glabrata*. We conducted a *in silico* evaluation of pharmaceutical properties, there is an assurance that they can be proposed as new drugs against IC. This evaluation *in silico* was carried out using the SwissADME tool. The pharmacokinetic characteristics of FE1 to FE3 evaluated were: LIPO: liposolubility, XLOGP3 between −0.7 y +5.0; SIZE: molecular weight between 150 and 500 g/mol; POLAR: polarity, TPSA between 20 Å and 130 Å; INSOLU: insolubility, log S not higher than 6; INSATU: insaturation, carbon fractions in the sp^3^ hybridization no lower than 0.25; FLEX: flexibility, no more than 9 rotating links. The *in silico* prediction by ADME analysis (absorption, distribution, metabolism and elimination) of FE1 to FE3 showed that all the compounds presented favorable pharmacokinetic properties (Fig. 3).

**Fig. 4:**
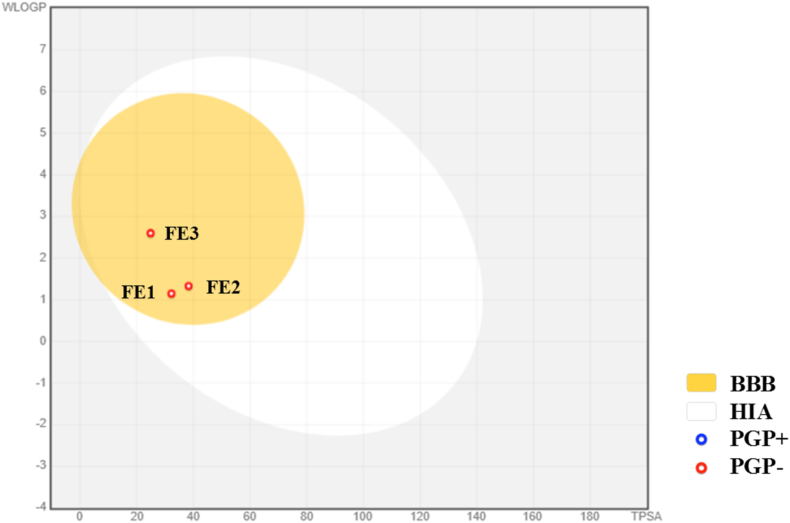
**Boiled-egg diagram of FE1 to FE3.** It shows the ability of the chemical molecule to penetrate the gastrointestinal tract (HIA) and the blood-brain barrier (BBB) according to the lipophilic (WLOGP) and the topologic polar area surface (TPSA).

Additionally, the boiled-egg diagram shows that FE1, FE2, and FE3 were able to penetrate the BBB (Fig. 4) [73]. This result shows the probability of permeability of the chemical molecules FE1 to FE3. However, even when an *in silico* analysis helps to know a primary approximation of the mechanism of permeability's of the chemical molecules as a drug, the efficacy of treatment against infections depends on certain factors, such as the interaction of the drug with the therapeutic target, the resistance of the microorganism and the capacity of the antifungal to reach optimal concentrations in various anatomical sites of the human.

Once the chemical molecules are chosen against these two proteins, the ligands are analyzed through docking, considering their contact print, as well as the best grading for the favorable interactions. In this way, the ligands that presented greater negative energies were chosen for the subsequent studies. The calculation of the affinity values (kcal/mol) in the interaction of the seven chemical molecules (FE1 to FE3) with Fba1 or Pk were obtained with the AutoDock Vina program. The docking study with the various 3D structures corresponding to Fba1 and Pk of *C. glabrata* was established with a search space of the dimensions indicated in the Materials and Methods. With these coordinates (Table 1), free energy of negative binding values was obtained.

**Table 1:** Energy of affinity (kcal/mol) resulting from the molecular coupling among the proteins of *C. glabrata* and the various compounds.

Ligand	Fba1	Pk
FE1	−5.0	−4.5
FE2	−5.1	−5.0
FE3	−5.7	−5.4

It was determined that there is no significant difference in the three molecules (FE1-FE3) evaluated (p value: 0.1544), given the theoretical scores of the joining of the target proteins of the study (protein-ligand). However, FE3 compound is the one that received the highest score in the interaction with the two proteins (Table 1) because the lowest value is considered the best in the formation of the ligand-protein complex. In the Fba1-FE3 interaction, an energy level of activity was obtained of −5.7 kcal/mol, and for the Pk-FE3, it was −5.4 kcal/mol, data that shows that possibly FE3 is the better ligand against Fba1 and Pk of *C. glabrata*. To evaluate whether the chemical molecule FE3 is effectively the one with the greatest effect *in vitro* against *C. glabrata,* we conducted a susceptibility test with all three molecules (FE1 to FE3). In the susceptibility assay, we used nystatin as the control antifungal, which is widely reported to be the drug against this pathogen [74].

**Fig. 5:**
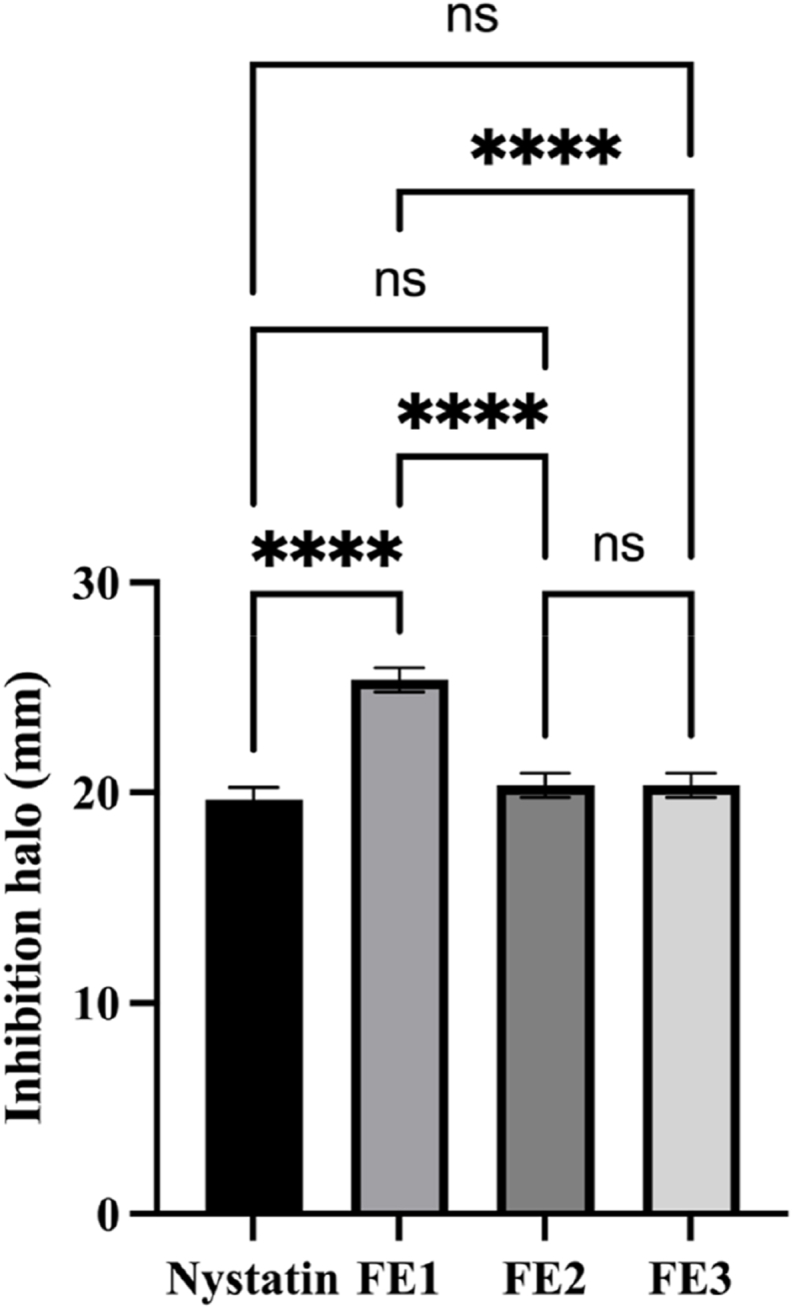
**Evaluation of the susceptibility of the FE1 to FE3 chemical molecules against *C. glabrata*.** Nystatin was the control antifungal used. The experiment was carried out in triplicate. Statistical analysis shows that there are significant differences (∗∗∗∗p value: <0.0001) and no significant differences (ns).

The susceptibility assays show that *C. glabrata* presented greater susceptibility to the chemical molecule FE1 regarding the commercial antifungal nystatin (Fig. 5). With the chemical molecule FE3, which obtained the best score by ligand docking to Fba1 and Pk the susceptibility study shows similar inhibition of *C. glabrata* to nystatin, but less than caused by FE1 (Fig. 5). The data obtained *in silico* and *in vitro* seem to indicate that in this case there is no correspondence in the totality between both, given that the three evaluated chemical molecules showed a good score for energy of affinity (Table 1) and any of these can be chosen as a good candidate against Fba1 and Pk. It was FE3 that presented the best score *in silico,* but the susceptibility assays showed the best ligand to be FE1. A possible explanation for these results may be due to i) the differences in the docking affinity values is given because FE1 or FE3 bind to different sites on the protein; ii) FE1, due to its chemical nature, when in interaction with the complete microorganism, is capable of binding more easily to the targets, while FE3 doesn't bind as easily to its targets. Another plausible explanation lies in the presence of a hydroxyl group in FE1, which confers greater polarity, likely improving aqueous solubility and bioavailability under physiological conditions. Enhanced solubility could facilitate better dispersion in the culture medium and improve compound uptake by fungal cells. In addition, in FE1, TPSA is higher and WLOGP is lower than in FE3. In the case of FE2, which shows intermediate values and a response equal to FE3, this could be due to the fact that FE2 contains a more extended and possibly more rigid structure (due to the presence of the carbon selenoyl group and the methyl ester), affecting the possible coupling.

These results seem highly interesting to us because although it is true that the analyses by docking are of great utility to virtually screening libraries for possible drugs against a certain illness in a very efficient way, and in the least time possible, it does not necessarily mean that the ligand that presents the best value *in silico*, will necessarily present the best functionality in *vitro* will (Fig. 5).

### 3.2 Fba1 and Pk of *C. glabrata* possess the characteristics of being therapeutic targets against IC

**Fig. 6:**
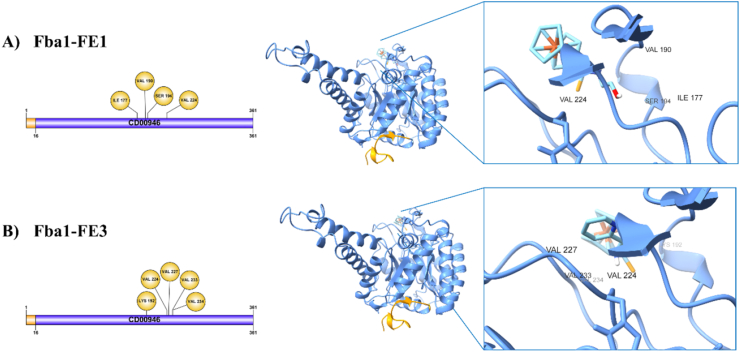
**Interaction between Fba1 of *C. glabrata* with the chemical molecules. A) FE1 or B) FE3.** The union of the Fba1-ligand is shown with the interaction of the specific amino acids.

For the purpose of finding out if the difference in the assays *in silico* with regard to the assays *in vitro* between FE1 and FE3 is due to the fact that they bind to different sites of Fba1 and Pk of *C. glabrata*, we evaluated the interaction of both chemical molecules with the two proteins, data that should favor the determination of which of the domains, specifically of the amino acids, can be considered to generate greater inhibition. The interactions observed in the FE1-Fba1 of *C. glabrata* are determined by hydrogen bonds between the serine residue 194 con the O of the OH group of FE1 at 3.01 Å, residue Ile177 with the H of the OH is at 2.78 Å, as well as Val190 with the H from NH is at 2.23 Å (Table 2, Fig. 6A).

**Table 2:** Affinity of FE1 and FE3 against Fba1 of *C. glabrata*.

Receptor	Ligand	Atom from ligand	Amino acid	Type of bond	Distance (Å)
Fba	FE1	O from OH	Ser194	C–H bond	3.01
*C. glabrata*		H from OH	Ile177	Hydrogen bond	2.78
		H from NH	Val190	Hydrogen bond	2.23
		C from COH	Val190	C–H bond	3.35
		Selenium	Val224	Alkyl	5.24
		Ferrocene	Val224	Alkyl	5.42
Fba	FE3	Pyridine	Val224	Pi-Alkyl	3.86
*C. glabrata*		Pyridine	Val227	Pi-Alkyl	5.44
		Pyridine	Val233	Pi-Alkyl	5.3
		Ferrocene	Val234	Alkyl	4.17
		Ferrocene	Lys192	Alkyl	5.27
		Selenium	Lys192	Alkyl	5.43

Another interaction found in this complex is the C–H between Val190 and the COH group of the FE1 at 3.35 Å. Two interactions of Val224 with selenium at 5.24 Å and ferrocene at 5.42 Å of FE1 were found, both corresponding to an interaction of the alkyl type (Table 2, Fig. 6A). For the complex Fba1-FE3, the implicated residues in their formation is given by three Pi-Alkyl links, the first between Val224 with the pyridine at 3.86 Å, the second between Val227 with pyridine at 5.44 Å and the third between the residue Val233 with the pyridine of FE3 at 5.3 Å. Along with these three interactions, this complex also had two interactions between Val234 and Lys192 with the ferrocene of FE3 at 4.17 and 5.27 Å, respectively, and one interaction between Lys192 and the selenium of FE3 (Table 2, Fig. 6B).

**Fig. 7:**
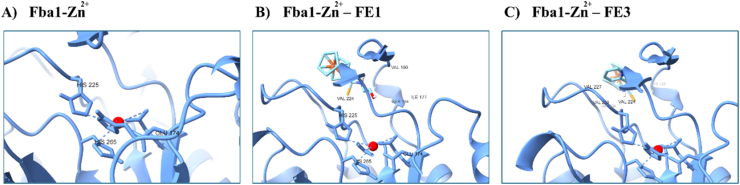
Interactions of Fba1 from *C. glabrata* with A) Zn^2+^, B) Zn^2+^-FE1 or C) Zn^2+^-FE3.

The results of the interactions of the Fba1-FE1 regarding the Fba1-FE3 complex show the difference in the interaction of the three amino acids of Fba1 with FE1 or four with FE3 and both ligands only share an interaction with Val224 (Table 2, Fig. 6). The Fba1 of *C. glabrata* belongs to the aldolases’ class II, which structurally stabilizes the carbonyl group through a divalent metallic cation, which in this case is Zn^2+^. In these enzymes, the metallic ion can act as a catalytic center, as a bond group to reunite the substrate with the enzyme, and as a stabilizing agent of the shape of the enzymatic protein. The Fba1 of *C. glabrata* is a homodimer, as are the aldolases belonging to class II [[75], [76], [77]]. In this sense, it has been observed that the Zn^2+^ of Fba1 of *C. glabrata* is found bound to the Glu174, His225 and His265 residues (Fig. 7A). When FE1 is joined to Fba1, it does so through the interactions with the Ser194, Ile177, Val190 and Val224 residues (Table 2, Fig. 6). In this interaction, it is possible that the nearness between Glu174 and Ile177, as well as of Val224 and His225, in a certain way, destabilizes the areas of contact with the catalytic zinc, or another option might be that the presence of the FE1 ligand favors the dislocation of the zinc charge and in this way FE1 inhibits Fbal (Fig. 7B). While in the Fba1-FE1 complex there are two interactions between His265 and Glu174 residues that could destabilize the catalytic zinc (Fig. 7B), in the Fba1-FE3 complex there are no close interactions (Fig. 7C), so an advantage in *in vitro* experiments could be attributed to FE1 over FE3 in the inhibition of Fba1.

**Fig. 8:**
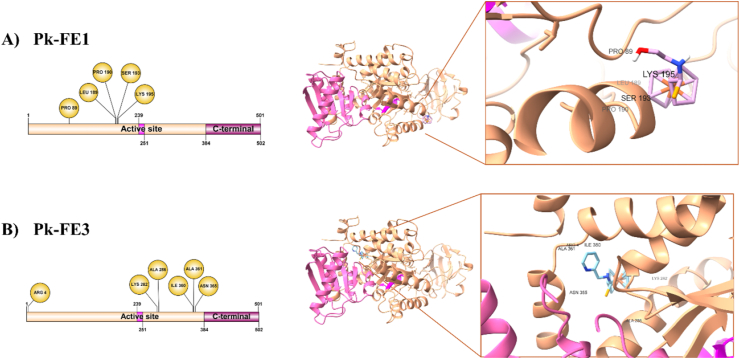
**Interaction between Pk of *C. glabrata* with the chemical molecules. A) FE1 or B) FE3.** The union of the Pk-ligand complex is shown with the interaction of the specific amino acids.

For the Pk, the same analysis with the chemical molecules FE1 and FE3 was carried out as in the case of Fba1. The amino acid residues that participated in the interaction of the complex formed by Pk-FE1 are Ser193 with the H from OH of the ligand at 2.07 Å, Pro89, Pro190, Leu189, and Lys195 with the ferrocene from FE1 (Table 3, Fig. 8A).

**Table 3:** Affinity of FE1 and FE3 against Pk of *C. glabrata*.

Receptor	Ligand	Atom from ligand	Aminoacid	Type of bond	Distance (Å)
Pk	FE1	H from OH	Ser193	Hydrogen bond	2.07
*C. glabrata*		Ferrocene	Pro89	Alkyl	5.28
		Ferrocene	Pro190	Alkyl	5.16
		Ferrocene	Leu189	Alkyl	4.36
		Selenium	Lys195	Alkyl	5.49
Pk	FE3	Pyridine	Ala361	C–H Bond	3.52
*C. glabrata*		Pyridine	Asn365	Hydrogen bond	3.36
		Pyridine	Arg4	Pi-Donor	3.21
		Pyridine	Ile360	Pi-Alkyl	4.83
		Ferrocene	Ala286	Alkyl	5.44
		Ferrocene	Lys282	Alkyl	3.87
		Ferrocene	Lys282	Alkyl	4.43

The Pk-FE3 complex is formed by the interactions of the Ala286 and Lys282 residues to the ferrocene of the ligand and the union between Arg4, Ile360, Ala361 and Asn365 with the pyridine (Table 3, Fig. 8B).

**Fig. 9:**
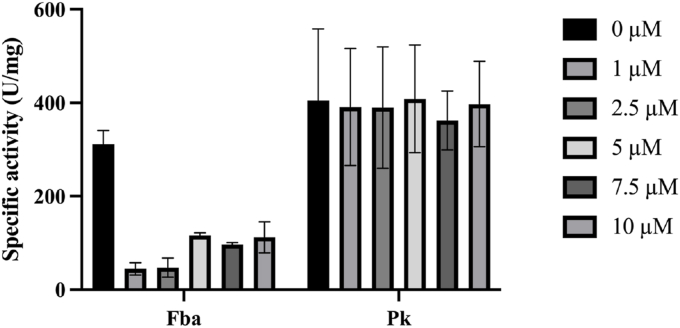
Specific activities of Fba1 and Pk of *C. glabrata* in the presence of 0, 1.0, 2.5, 5.0, 7.5 and 10.0 μM of FE1. Mean comparisons showed statistically significant differences for Fba1 (p < 0.0001), while no significant differences were observed for Pk (p = 0.9921).

Pk is an enzyme that generally exists as a homotetramer, presents allosteric regulation by binding to substrate, and reversible phosphorylation coordinating its activity according to the energy and synthesis demands of the cell [78,79]. Pk is made up of three domains A, B, and C [80]. Domain A generally includes the residues in positions 18–88 and 187–356. The residues of domain B are located in the 89–186 positions, while the C domain includes residue 400 and beyond, and it is in this domain where the site of the union to the effector was found, which is formed by the union residues to phosphate, a mobile domain, and the effector domain. The catalytic site is formed by residues that are found at the intersections of the A domain (residues 86–88) and B (residues 178–190). It has also been observed that the adjoining residues to the residues that form the catalytic site have an important functional role in the Pk [80]. The Pk-FE1 complex is formed by the interaction of the five residues of Pk with the chemical molecule FE1. Interestingly, the five residues are found very close to or inside the hinge that forms the catalytic site (Fig. 8A). In this way, when FE1 is placed in this position in Pk, the active site is inactivated. On the contrary, it was found that in the Pk-FE3 complex, which is formed by the interaction among six Pk residues with FE3, these are found far away from the residues that make up the hinge of the catalytic site (Fig. 8B). This data shows that the Pk of *C. glabrata* is an attractive therapeutic target against IC. Pk has not been considered as a target for the design of antifungals, but based on the results that are found, as well as on the work on other pathogens that have shown that this protein is a target in the treatment of other illnesses [[24], [25], [26]], we propose Pk as a therapeutic target. Additionally, for the purpose of corroborating that FE1 effectively inhibits or alters the enzymatic activity of Fba1 and Pk, we carried out assays of enzymatic activity for each of the two pure proteins. The purification of the two enzymes was carried out following the methodology described in the Materials and Methods. The overexpression and purity of the proteins were determined by electrophoretic analysis in a polyacrylamide gel at 12 % in denaturing conditions. No aggregation or proteolysis was observed, and the proteins presented the expected molecular weight. Enzymatic activity assays were performed on the purified proteins with the chemical molecule FE1 using kinetic spectrophotometry. The determination of the enzymatic activity of Fba1 measures the rate of DHAP beginning with d-fructose-1,6-bisphosphate. The DHAP was determined with the α-glycerophosphate dehydrogenase (GDH) in the presence of NADH_2_. The enzymatic activity of Pk was carried out by indirect assay, where the pyruvate formed by the reaction catalyzed by Pk from PEP and ADP is reduced to lactate, requiring NADH in the second reaction of lactate dehydrogenase. The obtained for Fba1 in the presence of various concentrations of FE1 show that this chemical molecule caused a significant effect on the enzymatic activity of Fba1 (Fig. 9). This data correlates with the results found in the susceptibility assay (Fig. 5) and the analysis of the Fba1 residues that participated in the interaction with FE1 (Fig. 6), which implies why Fba1 should be considered an important potential therapeutic target.

While the effect of FE1 on Pk was not significant compared to the control, a minimally inhibitory effect was found for this protein (Fig. 9). This suggests that the inhibition mechanism of FE1 on Pk is minor compared to the effect on Fba1 independently of the results of the molecular dockings obtained *in silico*. This result suggests that FE1 is a multitarget chemical molecule against IC, and when it interacts with the entire microorganism, on the one hand, it inhibits Fba1, but it also might generate free radicals via the Fenton reaction.

These results together show that the value obtained for the affinity energy *in silico* is a good parameter to screen the ligands with a higher score to inhibit therapeutic targets. However, in the near future, it might be necessary to validate with crystallographic analysis if all these chemical and experimental predictions are supported by X-ray crystal structures, which are not all available. On the other hand, when carrying out the verification experiment, it was determined that FE1 is an adequate ligand to inhibit Fba1 but not Pk.

Taking into consideration the above and the objective of obtaining potential new drugs, it is important to determine the safety of the selected molecules, so, complementing the pharmacokinetic profile, the toxicological prediction of the compounds FE1-FE3 was carried out (Table 4).

**Table 4:** Toxicity analysis of FE1-FE3 molecules.

Model name	FE1	FE2	FE3
AMES toxicity	No	No	No
Max. tolerated dose (human) [log mg/kg/day]	0.295	0.163	0.036
hERG I inhibitor	No	No	No
hERG II inhibitor	No	No	No
Oral Rat Acute Toxicity (LD50) [mol/kg]	2.503	2.636	2.651
Hepatotoxicity	No	No	Yes

Data obtained with the pkCSM server show that the three FE1-FE3 molecules are not potential mutagenic agents. In addition, they are also shown not to be cardiotoxic because they do not inhibit hERG (human ether-go-go gene) encoded potassium channels, which is important because inhibition of these channels is a cause of the occurrence of acquired long QT syndrome that can lead to fatal ventricular arrhythmia [81]. It was also determined that the molecules have no hepatotoxic effect, except for FE3, however, as mentioned, the best drug candidate molecule is FE1, and this analysis demonstrates that it has the best physicochemical, pharmacokinetic and toxicological characteristics.

Based on these conclusions, it was decided to perform a cytotoxicity assay to confirm the safety profile of FE1, as well as to determine the minimum inhibitory concentration (MIC) of the molecule with *Candida glabrata*. The results showed an IC50 of 4.34 ± 0.17 μM for the HaCaT cell line (human keratinocytes) and an IC50 of 6.63 ± 0.05 μM for COS-7 (monkey kidney fibroblasts). While the minimum inhibitory concentration of FE1 with *Candida glabrata* is 1 μg/mL, equivalent to 2.97 μM, thus, there is a reduced therapeutic margin, but with potential to be evaluated in an *in vivo* model.

### 3.3 Conclusions

The repositioning of drugs is an alternative that allows for the identification of candidate chemical molecules against IFIs in a more efficient way, since it reduces the time in which to evaluate and place drugs on the market. In the present work, considering a group of tools *in silico* and experiments *in vitro,* it was possible to identify the best candidate molecule as a possible antifungal for the treatment of IC caused by *C. glabrata*. This repositioning of the chosen chemical molecule as a possible new antifungal could be used in the near future as monotherapy or polytherapy against IC and in this way, contribute to a better quality of life for patients, as well as reducing the mortality rates from this infection.

## CRediT authorship contribution statement

**Edson E. Maqueda-Cabrera:** Writing – review & editing, Software, Methodology, Investigation, Formal analysis. **Alejandro Castillo-Baltazar:** Writing – review & editing, Methodology, Investigation. **Nancy A. Vázquez-López:** Writing – review & editing, Methodology, Investigation. **Maritza Almanza-Villegas:** Writing – review & editing, Methodology, Investigation. **María Teresa Ramírez-Apan:** Writing – review & editing, Methodology, Investigation. **M. Carmen Ortega-Alfaro:** Writing – review & editing, Validation, Methodology, Investigation. **José G. López-Cortés:** Writing – review & editing, Validation, Methodology, Investigation. **Abel Moreno:** Writing – original draft, Validation, Supervision, Methodology, Investigation, Formal analysis, Conceptualization. **Mayra Cuéllar-Cruz:** Writing – original draft, Validation, Supervision, Resources, Project administration, Methodology, Investigation, Funding acquisition, Formal analysis, Conceptualization.

## Funding

This work was carried out with the financial support granted to M. Cuéllar-Cruz by Project No. CF2019-39216 from the *Consejo Nacional de Ciencias, Humanidades y Tecnologías* (CONAHCYT) and *Proyecto-Institucional-UGTO*-005/2024 from *Universidad de Guanajuato*, México. One of the authors (AM) acknowledges the support of DGAPA-UNAM Project No. IN206125 and Project No. CBF-2025-I-183 from the *Secretaria de Ciencia, Tecnología, Humanidades e Innovación* (SECIHTI), Mexico.

## Declaration of competing interest

The authors declare that they have no known competing financial interests or personal relationships that could have appeared to influence the work reported in this paper.

## References

[1] Casalini G., Giacomelli A., Antinori S. (2024). The WHO fungal priority pathogens list: a crucial reappraisal to review the prioritisation. Lancet Microbe.

[2] Wiederhold N.P. (2022). Emerging fungal infections: new species, new names, and antifungal resistance. Clin. Chem..

[3] Suleyman G., Alangaden G.J. (2021). Nosocomial fungal infections: epidemiology, infection control, and prevention. Infect. Dis. Clin..

[4] Denning D.W. (2024). Global incidence and mortality of severe fungal disease. Lancet Infect. Dis..

[5] Goemaere B., Lagrou K., Spriet I., Hendrickx M., Becker P. (2018). Clonal spread of *Candida glabrata* bloodstream isolates and fluconazole resistance affected by prolonged exposure: a 12-Year single-center Study in Belgium, antimicrob. Agents. Chemother..

[6] Borman A.M., Johnson E.M. (2021). Name changes for fungi of medical importance. J. Clin. Microbiol..

[7] Takashima M., Sugita T. (2022). Taxonomy of pathogenic yeasts *candida, cryptococcus, malassezia*, and *Trichosporon*. Med. Mycol. J..

[8] Katsipoulaki M., Stappers M.H.T., Malavia-Jones D., Brunke S., Hube B., N.A.R. (2024). *Candida albicans* and *Candida glabrata*: global priority pathogens. Microbiol. Mol. Biol. Rev..

[9] Frías-De-león M.G., Hernández-Castro R., Conde-Cuevas E., García-Coronel I.H., Vázquez-Aceituno V.A., Soriano-Ursúa M.A., Farfán-García E.D., Ocharán-Hernández E., Rodríguez-Cerdeira C., Arenas R., Robledo-Cayetano M., Ramírez-Lozada T., Meza-Meneses P., Pinto-Almazán R., Martínez-Herrera E. (2021). *Candida glabrata* antifungal resistance and virulence factors, a perfect pathogenic combination. Pharmaceutics.

[10] Roetzer A., Klopf E., Gratz N., Marcet-Houben M., Hiller E., Rupp S., Gabaldón T., Kovarik P., Schüller C. (2011). Regulation of *Candida glabrata* oxidative stress resistance is adapted to host environment. FEBS Lett..

[11] Cuéllar-Cruz M., Briones-Martin-del-Campo M., Cañas-Villamar I., Montalvo-Arredondo J., Riego-Ruiz L., Castaño I., De Las Peñas A. (2008). High resistance to oxidative stress in the fungal pathogen *Candida glabrata* is mediated by a single catalase, Cta1p, and is controlled by the transcription factors Yap1p, Skn7p, Msn2p, and Msn4p. Eukaryot. Cell.

[12] Lim C.S.Y., Rosli R., Seow H.F., Chong P.P. (2012). *Candida* and invasive candidiasis: back to basics. Eur. J. Clin. Microbiol. Infect. Dis..

[13] Hassan Y., Chew S.Y., Than L.T.L. (2021). *Candida glabrata*: pathogenicity and resistance mechanisms for adaptation and survival. J. Fungi.

[14] Leajan Chaffin W., José Luis López-Ribot, Casanova M., Gozalbo D., Martínez José P. (1998). Cell Wall and secreted proteins of *Candida albicans*: identification, function, and expression. Microbiol. Mol. Biol. Rev..

[15] Caro L.H.P., Tettelin H., Vossen J.H., Ram A.F.J., Van Den Ende H., Klis F.M. (1997). *In silico* identification of glycosyl-phosphatidylinositol-anchored plasma-membrane and cell wall proteins of *Saccharomyces cerevisiae*. Yeast.

[16] Ecker M., Deutzmann R., Lehle L., Mrsa V., Tanner W. (2006). Pir proteins of *Saccharomyces cerevisiae* are attached to β-1,3-glucan by a new protein-carbohydrate linkage. J. Biol. Chem..

[17] Toh-E A., Oguchi T., Matsui Y., Yasunaga S., Nisogi H., Tanaka K. (1993). Three yeast genes, PIR1, PIR2 and PIR3, containing internal tandem repeats, are related to each other, and PIR1 and PIR2 are required for tolerance to heat shock. Yeast.

[18] Insenser M.R., Hernáez M.L., Nombela C., Molina M., Molero G., Gil C. (2010). Gel and gel-free proteomics to identify *Saccharomyces cerevisiae* cell surface proteins. J. Proteonomics.

[19] Ramírez-Quijas M.D., López-Romero E., Cuéllar-Cruz M. (2015). Proteomic analysis of cell wall in four pathogenic species of *Candida* exposed to oxidative stress. Microb. Pathog..

[20] Serrano-Fujarte I., López-Romero E., Cuéllar-Cruz M. (2016). Moonlight-like proteins of the cell wall protect sessile cells of *Candida* from oxidative stress. Microb. Pathog..

[21] Swoboda R.K., Bertram G., Hollander H., Greenspan D., Greenspan J.S., Gow N.A., Gooday G.W., Brown A.J. (1993). Glycolytic enzymes of *Candida albicans* are nonubiquitous immunogens during candidiasis. Infect. Immun..

[22] Medrano-Díaz C.L., Vega-González A., Ruiz-Baca E., Moreno A., Cuéllar-Cruz M. (2018). Moonlighting proteins induce protection in a mouse model against *Candida* species. Microb. Pathog..

[23] qiu Li F., fang Ma C., ning Shi L., fen Lu J., Wang Y., Huang M., qian Kong Q. (2013). Diagnostic value of immunoglobulin G antibodies against *Candida* enolase and fructose-bisphosphate aldolase for candidemia. BMC Infect. Dis..

[24] Amiri-Dashatan N., Rezaei-Tavirani M., Ranjbar M.M., Koushki M., Mousavi Nasab S.D., Ahmadi N. (2021). Discovery of Novel Pyruvate kinase inhibitors against *Leishmania* major among FDA approved drugs through System biology and molecular docking approach. Turk. J. Pharm. Sci..

[25] Akunuri R., Unnissa T., Vadakattu M., Bujji S., Mahammad Ghouse S., Madhavi Yaddanapudi V., Chopra S., Nanduri S. (2022). Bacterial pyruvate kinase: a new potential target to combat drug-resistant *Staphylococcus aureus* infections. ChemistrySelect.

[26] Zoraghi R., See R.H., Axerio-Cilies P., Kumar N.S., Gong H., Moreau A., Hsing M., Kaur S., Swayze R.D., Worrall L., Amandoron E., Lian T., Jackson L., Jiang J., Thorson L., Labriere C., Foster L., Brunham R.C., McMaster W.R., Finlay B.B. (2011). Identification of pyruvate kinase in methicillin-resistant *Staphylococcus aureus* as a novel antimicrobial drug target. Antimicrob. Agents Chemother..

[27] Fisher M.C., Alastruey-Izquierdo A., Berman J., Bicanic T., Bignell E.M., Bowyer P., Bromley M., Brüggemann R., Garber G., Cornely O.A., Gurr S.J., Harrison T.S., Kuijper E., Rhodes J., Sheppard D.C., Warris A., White P.L., Xu J., Zwaan B., Verweij P.E. (2022). Tackling the emerging threat of antifungal resistance to human health. Nat. Rev. Microbiol..

[28] Lu H., Hong T., Jiang Y., Whiteway M., Zhang S. (2023). Candidiasis: from cutaneous to systemic, new perspectives of potential targets and therapeutic strategies. Adv. Drug Deliv. Rev..

[29] Eliaš D., Gbelská Y. (2022). *Candida glabrata* - basic characteristics, virulence, treatment, and resistance. Epidemiol. Mikrobiol. Imunol..

[30] Namid A., Rani N.A., Robin T.B., Mashur M.N., Shovo M.M.I., Prome A.A., Sultana S., Nazneen Akhand M.R. (2024). Designing a multi-epitope subunit vaccine against *Toxoplasma gondii* through reverse vaccinology approach. Mol. Biochem. Parasitol..

[31] Huerta-Cepas J., Szklarczyk D., Heller D., Hernández-Plaza A., Forslund S.K., Cook H., Mende D.R., Letunic I., Rattei T., Jensen L.J., von Mering C., Bork P. (2019). eggNOG 50: a hierarchical, functionally and phylogenetically annotated orthology resource based on 5090 organisms and 2502 viruses. Nucleic Acids Res..

[32] Sievers F., Higgins D.G. (2018). Clustal Omega for making accurate alignments of many protein sequences. Protein Sci..

[33] Madeira F., Madhusoodanan N., Lee J., Eusebi A., Niewielska A., Tivey A.R.N., Lopez R., Butcher S. (2024). The EMBL-EBI Job Dispatcher sequence analysis tools framework in 2024. Nucleic Acids Res..

[34] Paysan-Lafosse T., Blum M., Chuguransky S., Grego T., Pinto B.L., Salazar G.A., Bileschi M.L., Bork P., Bridge A., Colwell L., Gough J., Haft D.H., Letunić I., Marchler-Bauer A., Mi H., Natale D.A., Orengo C.A., Pandurangan A.P., Rivoire C., Sigrist C.J.A. (2023). InterPro in 2022. Nucleic Acids Res..

[35] Abramson J., Adler J., Dunger J., Evans R., Green T., Pritzel A., Ronneberger O., Willmore L., Ballard A.J., Bambrick J., Bodenstein S.W., Evans D.A., Hung C.C., O'Neill M., Reiman D., Tunyasuvunakool K., Wu Z., Žemgulytė A., Arvaniti E., Beattie C. (2024). Accurate structure prediction of biomolecular interactions with AlphaFold 3. Nature.

[36] Meng E.C., Goddard T.D., Pettersen E.F., Couch G.S., Pearson Z.J., Morris J.H., Ferrin T.E. (2023). UCSF ChimeraX: tools for structure building and analysis, protein. Science.

[37] Colovos C., Yeates T.O. (1993). Verification of protein structures: patterns of nonbonded atomic interactions, protein. Science..

[38] Bowie J.U., Lüthy R., Eisenberg D. (1991). A method to identify protein sequences that fold into a known three-dimensional structure. Science.

[39] Laskowski R.A., MacArthur M.W., Moss D.S., Thornton J.M. (1993). PROCHECK: a program to check the stereochemical quality of protein structures. J. Appl. Crystallogr..

[40] Fiser A., Sali A. (2003). ModLoop: automated modeling of loops in protein structures. Bioinformatics.

[41] Land H., Humble M.S. (2018). Methods in Molecular Biology.

[42] Hanwell M.D., Curtis D.E., Lonie D.C., Vandermeersch T., Zurek E., Hutchison G.R. (2012). SOFTWARE Open Access Avogadro: an advanced semantic chemical editor, visualization, and analysis platform. J. Cheminf..

[43] Trott O., Olson A.J. (2010). AutoDock Vina: improving the speed and accuracy of docking with a new scoring function, efficient optimization, and multithreading. J. Comput. Chem..

[44] Dallakyan S., Olson A.J. (2015). Small-molecule library screening by docking with PyRx. Methods Mol. Biol..

[45] Waterborg J.H., Matthews H.R., Walker J.M. (1994). Basic Protein and Peptide Protocols.

[46] Gutiérrez-Hernández A.I., López-Cortés J.G., Ortega-Alfaro M.C., Ramírez-Apan M.T., De Jesús Cázares-Marinero J., Toscano R.A. (2012). Ferrocenylselenoamides: synthesis, characterization and cytotoxic properties. J. Med. Chem..

[47] Ramírez-Gómez A., Gutiérrez A., Alvarado M., Toscano R., Ortega-Alfaro M., López-Cortés J. (2020). Selenoamides as powerful scaffold to build imidazo[1,5-a]pyridines using a grinding protocol. J. Organomet. Chem..

[48] Daina A., Michielin O., Zoete V. (2017). SwissADME: a free web tool to evaluate pharmacokinetics, drug-likeness and medicinal chemistry friendliness of small molecules. Sci. Rep..

[49] Pires D.E.V., Blundell T.L., Ascher D.B. (2015). pkCSM: predicting small-molecule pharmacokinetic and toxicity properties using graph-based signatures. J. Med. Chem..

[50] Warawdekar U.M., Kathuria D., Mande P., Mulherkar R. (2006). Optimization of the sulpharhodamine B (SRB) assay for the in vitro HSV-tk suicide gene therapy protocol proposed for the treatment of head and neck cancers. Cancer Epidemiol. Biomarkers Prev..

[51] CLSI (2004).

[52] Pearson W.R. (2013). An introduction to sequence similarity (“Homology”) searching. Curr. Protoc. Bioinformatics.

[53] Jumper J., Evans R., Pritzel A., Green T., Figurnov M., Ronneberger O., Tunyasuvunakool K., Bates R., Žídek A., Potapenko A., Bridgland A., Meyer C., Kohl S.A.A., Ballard A.J., Cowie A., Romera-Paredes B., Nikolov S., Jain R., Adler J., Back T. (2021). Applying and improving AlphaFold at CASP14, proteins: structure. Function and Bioinformatics.

[54] Zoraghi R., Worrall L., See R.H., Strangman W., Popplewell W.L., Gong H., Samaai T., Swayze R.D., Kaur S., Vuckovic M., Finlay B.B., Brunham R.C., McMaster W.R., Davies-Coleman M.T., Strynadka N.C., Andersen R.J., Reiner N.E. (2011). Methicillin-resistant *Staphylococcus aureus* (MRSA) pyruvate kinase as a target for bis-indole alkaloids with antibacterial activities. J. Biol. Chem..

[55] Tulloch L.B., Morgan H.P., Hannaert V., Michels P.A.M., Fothergill-Gilmore L.A., Walkinshaw M.D. (2008). Sulphate removal induces a major conformational change in *Leishmania mexicana* Pyruvate Kinase in the crystalline State. J. Mol. Biol..

[56] Jurica M.S., Mesecar A., Heath P.J., Shi W., Nowak T., Stoddard B.L. (1998). The allosteric regulation of pyruvate kinase by fructose-1,6-bisphosphate. Structure.

[57] Larsen T.M., Laughlin L.T., Holden H.M., Rayment I., Reed G.H. (1994). Structure of rabbit muscle pyruvate Kinase complexed with Mn^2+^, K^+^, and pyruvate. Biochemistry.

[58] Soriano A., Honore P.M., Puerta-Alcalde P., Garcia-Vidal C., Pagotto A., Gonçalves-Bradley D.C., Verweij P.E. (2023). Invasive candidiasis: current clinical challenges and unmet needs in adult populations. J. Antimicrob. Chemother..

[59] Zhang J., Wang Z., Gai C., Yang F., Yun X., Jiang B., Zou Y., Meng Q., Zhao Q., Chai X. (2024). Design, synthesis, evaluation and optimization of novel azole analogues as potent antifungal agents. Bioorg. Med. Chem..

[60] Lass-Flörl C., Kanj S.S., Govender N.P., Thompson G.R., Ostrosky- Zeichner L., Govrins M.A. (2024). Invasive candidiasis. Nat. Rev. Dis. Primers.

[61] Plewczynski D., Łażniewski M., Von Grotthuss M., Rychlewski L., Ginalski K. (2011). VoteDock: consensus docking method for prediction of protein–ligand interactions. J. Comput. Chem..

[62] Lill M., Kortagere S. (2013). *In Silico* Models for Drug Discovery.

[63] Lloyd M.D. (2020). High-Throughput screening for the discovery of enzyme inhibitors. J. Med. Chem..

[64] Papapetropoulos A., Szabo C. (2018). Inventing new therapies without reinventing the wheel: the power of drug repurposing. Br. J. Pharmacol..

[65] Poroikov V., Druzhilovskiy D., Roy Kunal (2019). Drug Repositioning: New Opportunities for Older Drugs.

[66] Naveja J. de J., Duenas-Gonzalez A., Medina-Franco J., Medina-Franco José L. (2016). Drug Repurposing for Epigenetic Targets Guided by Computational Methods.

[67] Wall G., Lopez-Ribot Jose L. (2020). Screening repurposing libraries for identification of drugs with novel antifungal activity, antimicrob. Agents. Chemother..

[68] Sharma B., Kumar V. (2021). Has ferrocene really delivered its role in accentuating the bioactivity of organic scaffolds?. J. Med. Chem..

[69] Anusionwu C.G., Fonkui T.Y., Oselusi S.O., Egieyeh S.A., Aderibigbe B.A., Mbianda X.Y. (2024). Ferrocene-bisphosphonates hybrid drug molecules: *in vitro* antibacterial and antifungal, in silico ADME, drug-likeness, and molecular docking studies, results. Chem.

[70] Vitaku E., Smith D.T., Njardarson J.T. (2014). Analysis of the structural diversity, substitution patterns, and frequency of nitrogen heterocycles among US FDA approved pharmaceuticals. J. Med. Chem..

[71] Ang C.W., Jarrad A.M., Cooper M.A., Blaskovich M.A.T. (2017). Nitroimidazoles: molecular fireworks that combat a broad spectrum of infectious diseases. J. Med. Chem..

[72] Sandoval-Chávez C., López-Cortés J.G., Gutiérrez-Hernández A.I., Ortega-Alfaro M.C., Toscano A., Alvarez-Toledano C. (2009). An expedient approach to ferrocenyl thioamides via Fischer carbenes. J. Organomet. Chem..

[73] Daina A., Zoete V. (2016). A BOILED-Egg to predict gastrointestinal absorption and brain penetration of small molecules. ChemMedChem.

[74] Sousa F., Nascimento C., Ferreira D., Reis S., Costa P. (2023). Reviving the interest in the versatile drug nystatin: a multitude of strategies to increase its potential as an effective and safe antifungal agent. Adv. Drug Deliv. Rev..

[75] Lorentzen E., Pohl E., Zwart P., Stark A., Russell R.B., Knura T., Hensel R., Siebers B. (2003). Crystal structure of an archaeal class I aldolase and the evolution of (βα)8 barrel proteins. J. Biol. Chem..

[76] Witke C., Gotz F. (1993). Cloning, sequencing, and characterization of the gene encoding the class I Fructose-1,6-Bisphosphate aldolase of *Staphylococcus carnosus*. J. Bacteriol..

[77] Götz F., Fischer S., Schleifer K.-H. (1980). Purification and characterisation of an unusually heat-stable and Acid/Base-Stable class I Fructose-1,6-bisphosphate aldolase from *Staphylococcus aureus*. Eur. J. Biochem..

[78] Knowles V.L., Smith C.S., Smith C.R., Plaxton W.C. (2001). Structural and regulatory properties of pyruvate kinase from the *Cyanobacterium synechococcus* PCC 6301. J. Biol. Chem..

[79] Enriqueta Muñoz Ma, Ponce E. (2003). Pyruvate kinase: current status of regulatory and functional properties. Comp. Biochem. Physiol., Part B: Biochem. Mol. Biol..

[80] Schormann N., Hayden K.L., Lee P., Banerjee S., Chattopadhyay D. (2019). An overview of structure, function, and regulation of pyruvate kinases, Protein. Science..

[81] Marchese Robinson R.L., Glen R.C., Mitchell J.B.O. (2011). Development and comparison of hERG blocker classifiers: assessment on different datasets yields markedly different results. Mol. Inform..

