# Supplementary material for: New insights on Drug's design against candidiasis on the fructose biphosphate aldolase (Fba1) and the pyruvate kinase (Pk) of Candida glabrata
Source: Biochem Biophys Rep. 2025 Jul 25;43:102175. doi: 10.1016/j.bbrep.2025.102175 (PMC12318263; doi:10.1016/j.bbrep.2025.102175)
Supplement: Multimedia component 1 [file mmc1.docx]

**New Insights on Drug’s Design against Candidiasis on the Fructose Biphosphate Aldolase (Fba1) and the Pyruvate Kinase (Pk) of *Candida glabrata***

**Edson E. Maqueda-Cabrera^1^, Alejandro Castillo-Baltazar^2^, Nancy A. Vázquez-López^1^, Maritza Almanza-Villegas^1^, María Teresa Ramírez-Apan^2^, M. Carmen Ortega-Alfaro^3^, José G. López-Cortés^2^, Abel Moreno^2*^ and Mayra Cuéllar-Cruz^1*^**

^1^*Departamento de Biología, División de Ciencias Naturales y Exactas, Campus Guanajuato, Universidad de Guanajuato, Noria Alta S/N, Col. Noria Alta, C.P. 36050, Guanajuato, Guanajuato, México.*

*^2^Instituto de Química, Universidad Nacional Autónoma de México, Av. Universidad 3000, Ciudad Universitaria, Ciudad de México, C.P. 04510. México*

*^3^Instituto de Ciencias Nucleares, Universidad Nacional Autónoma de México, Av. Universidad 3000, Ciudad Universitaria, Ciudad de México, C.P. 04510. México.*

^*^Corresponding Authors:

^1*^(M.C.C.) Departamento de Biología, División de Ciencias Naturales y Exactas, Campus Guanajuato, Universidad de Guanajuato, Noria Alta S/N, Col. Noria Alta, C.P. 36050, Guanajuato, Guanajuato, México. E-mail: [mcuellar@ugto.mx](mailto:mcuellar@ugto.mx).

^2*^(A.M.) Instituto de Química, Universidad Nacional Autónoma de México, Av. Universidad 3000, Ciudad Universitaria, Ciudad de México, 04510. México. E-mail: carcamo@unam.mx.

**SUPPORTING INFORMATION**


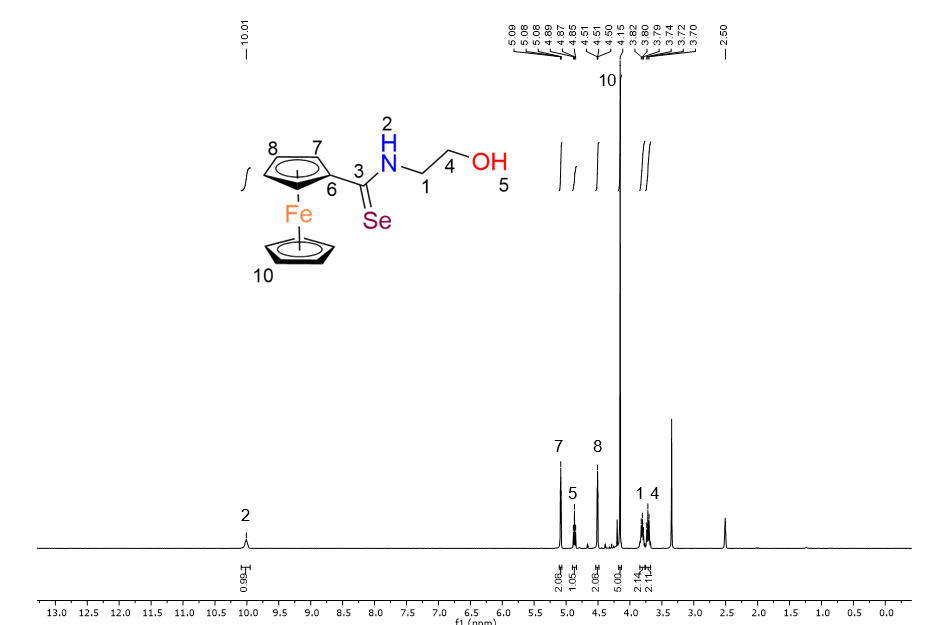


**Figure S1**. ^1^H NMR (300 MHz DMSO-*d*_6_) of compound **FE1**


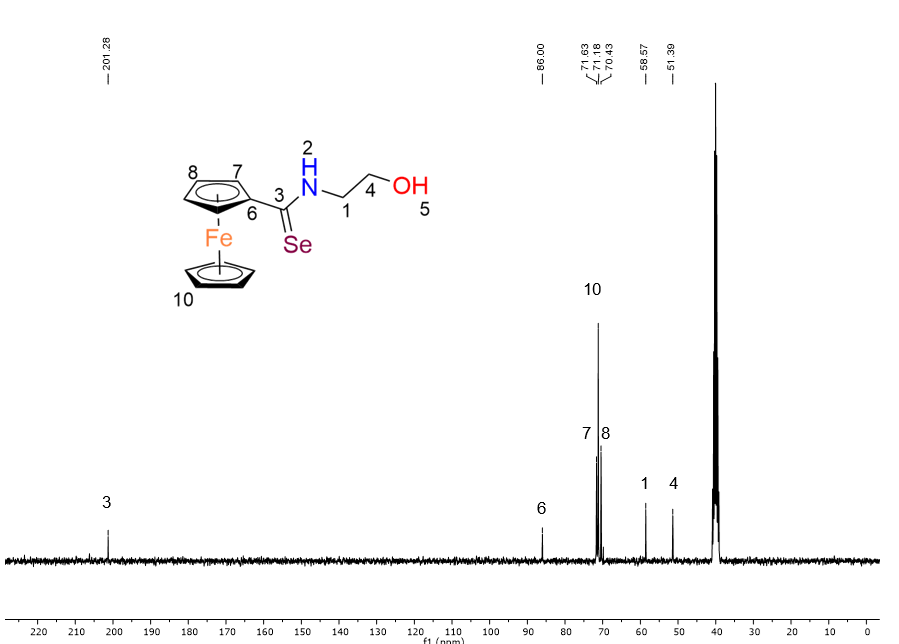


**Figure S2**. ^13^C NMR (75 MHz DMSO-*d*_6_) of compound **FE1**

**
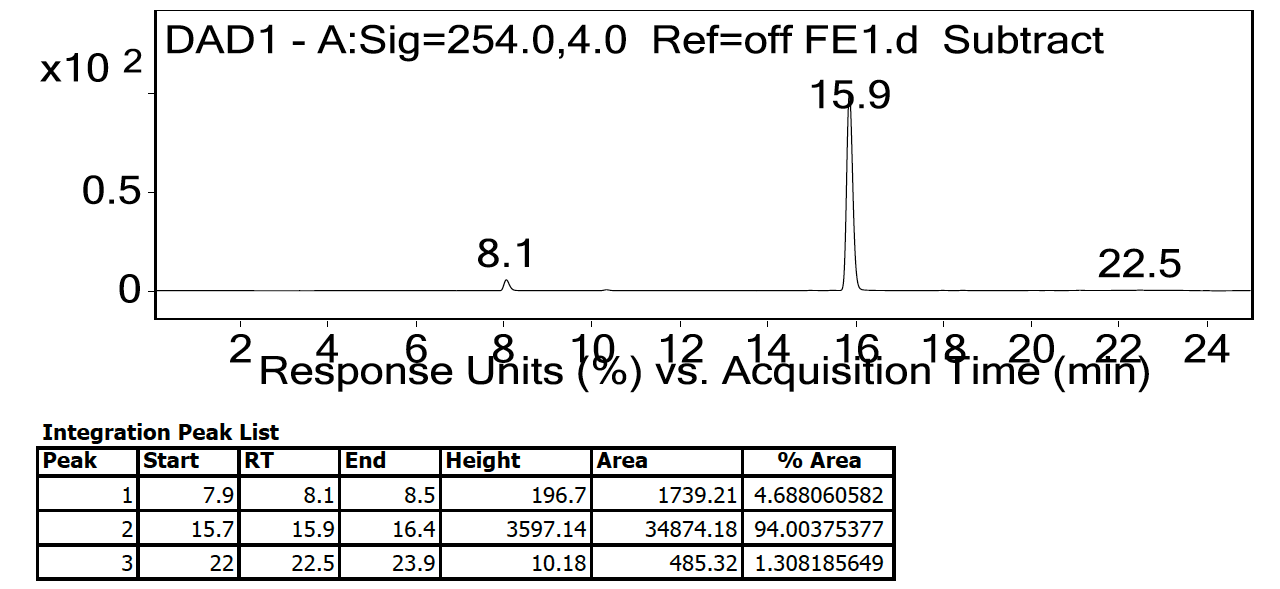
**

**Figure S3**. HPLC Chromatogram for **FE1**


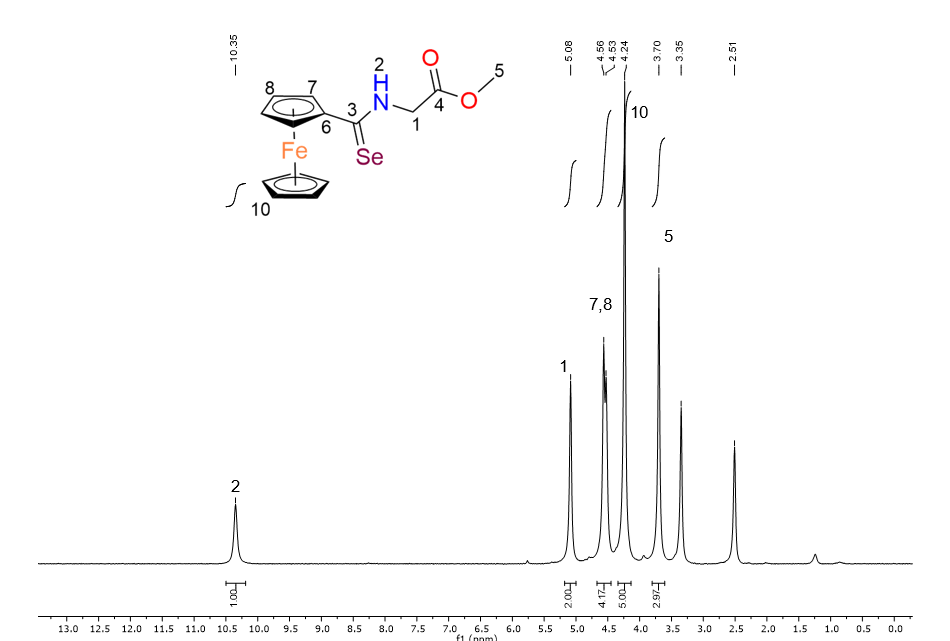


**Figure S4**. ^1^H NMR (300 MHz DMSO-*d*_6_) of compound **FE2**


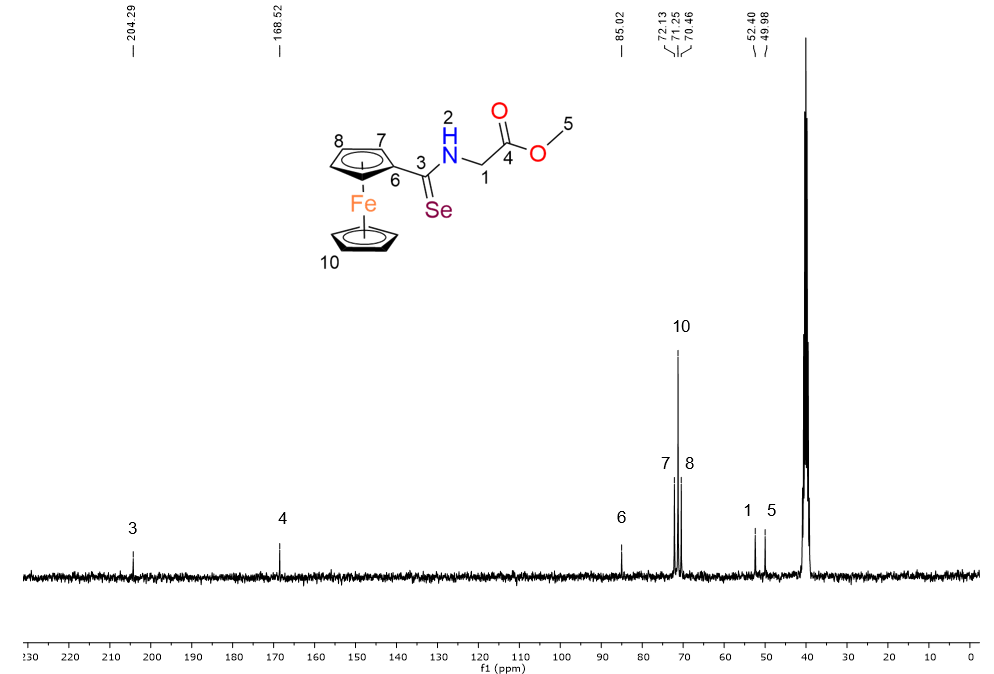


**Figure S5**. ^13^C NMR (75 MHz DMSO-*d*_6_) of compound **FE2**

**
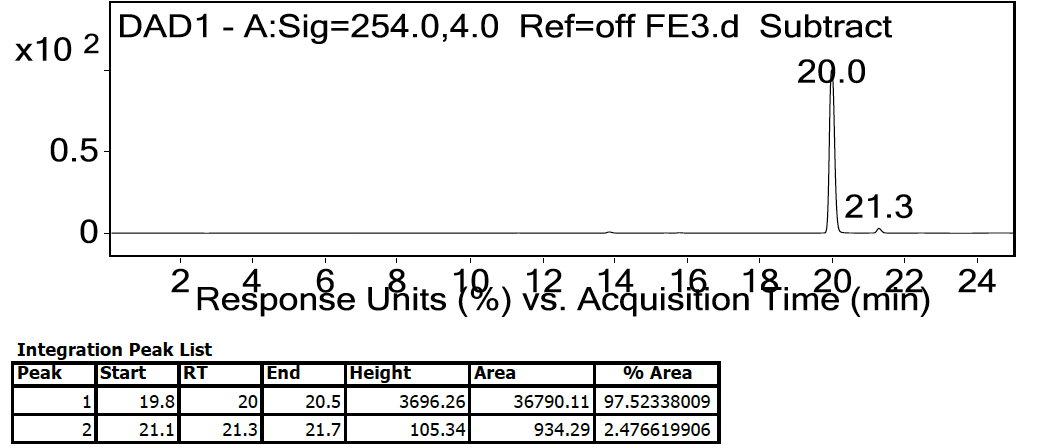
**

**Figure S6**. HPLC Chromatogram for **FE2**


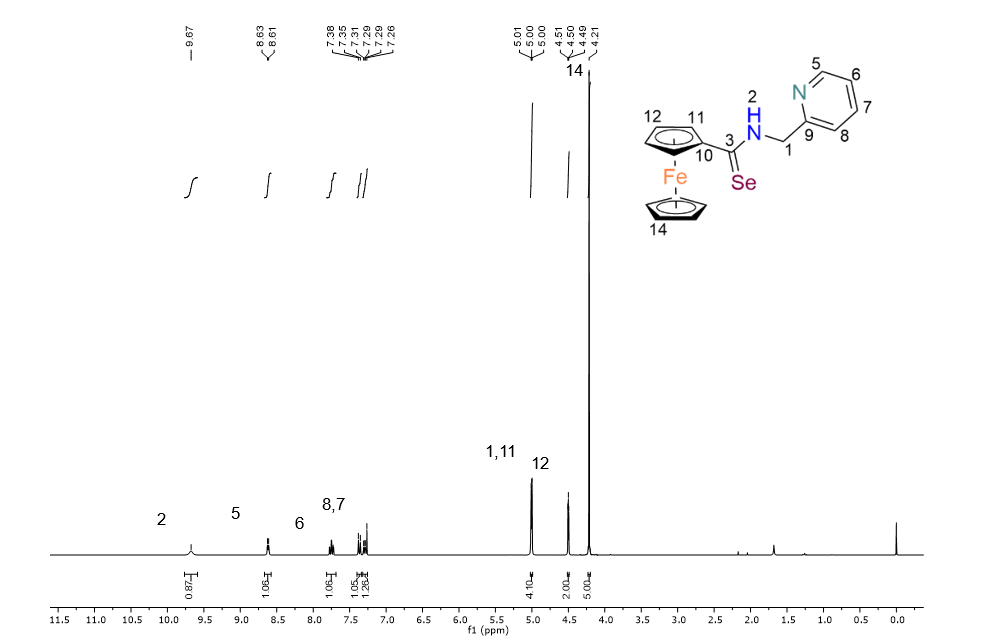


**Figure S7**. ^1^H NMR (300 MHz CDCl_3_) of compound **FE3**


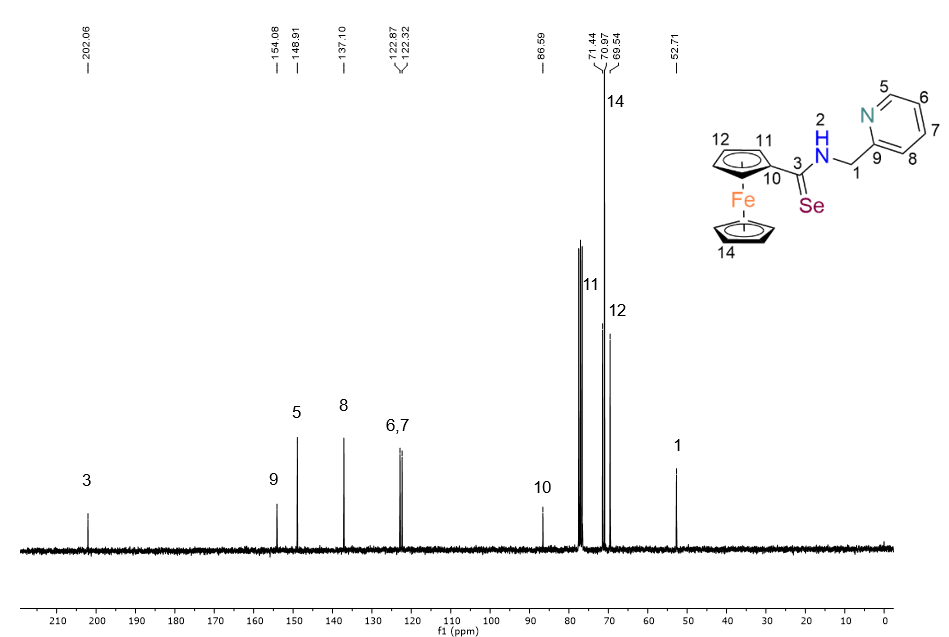


**Figure S8**. ^13^C NMR (75 MHz CDCl_3_) of compound **FE3**


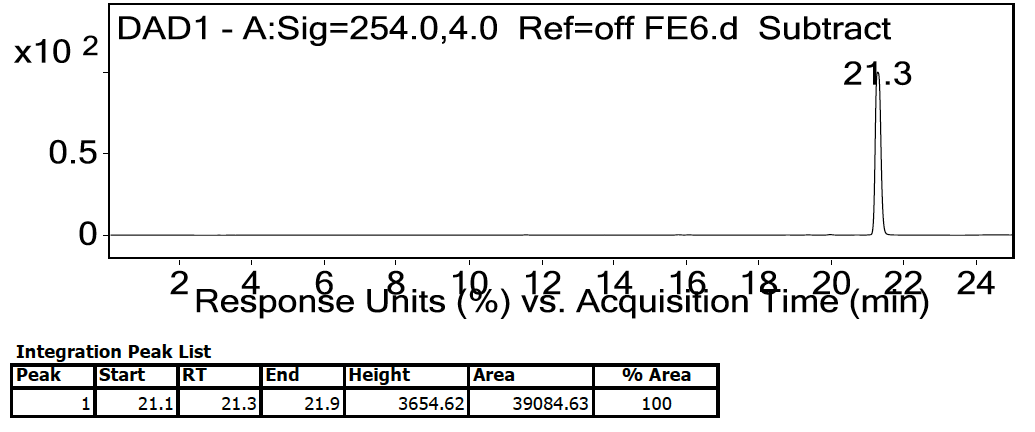


**Figure S9**. HPLC Chromatogram for **FE3**


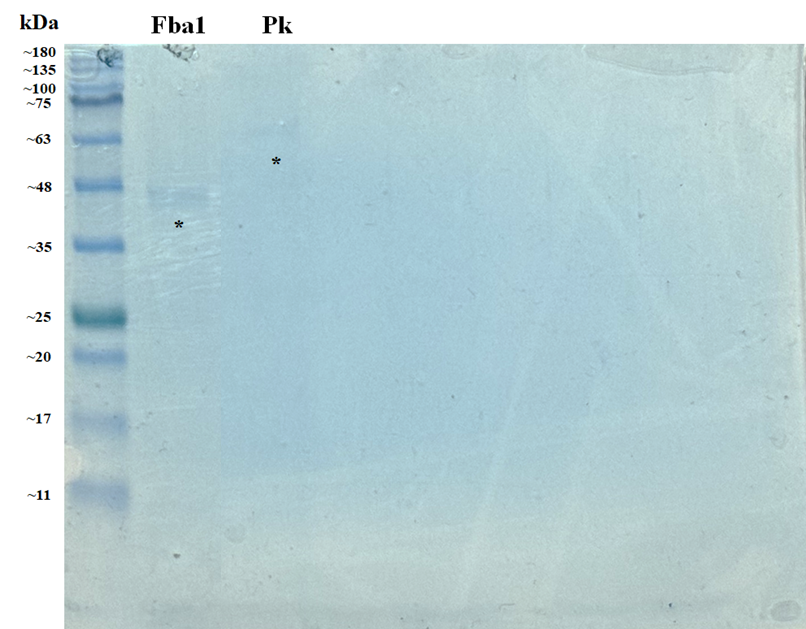


**Figure S10.** The overexpression and purity of the Fba1 and Pk proteins was determined by electrophoretic analysis in a polyacrylamide gel at 12% in denaturizing conditions
